# Metal-free formal synthesis of phenoxazine

**DOI:** 10.3762/bjoc.14.126

**Published:** 2018-06-20

**Authors:** Gabriella Kervefors, Antonia Becker, Chandan Dey, Berit Olofsson

**Affiliations:** 1 Department of Organic Chemistry, Arrhenius Laboratory, Stockholm University, SE-106-91 Stockholm, Sweden

**Keywords:** arylation, cyclization, diaryl ether, diaryliodonium salt, phenol

## Abstract

A transition metal-free formal synthesis of phenoxazine is presented. The key step of the sequence is a high-yielding *O*-arylation of a phenol with an unsymmetrical diaryliodonium salt to provide an *ortho*-disubstituted diaryl ether. This species was cyclized to acetylphenoxazine in moderate yield. The overall yield in the three-step sequence is 72% based on recovered diaryl ether. An interesting, unusually stable iodine(III) intermediate in the *O*-arylation was observed by NMR and could be converted to the product upon longer reaction time.

## Introduction

**Figure 1:**
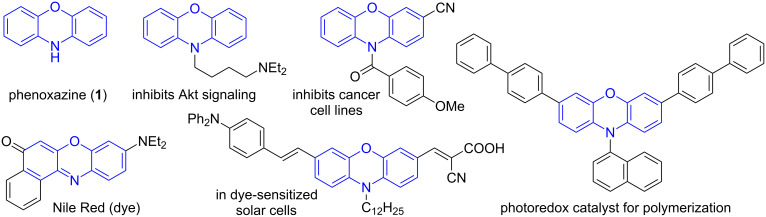
Compounds containing a phenoxazine moiety.

Phenoxazine (**1**) is a tricyclic compound consisting of an oxazine ring fused between two benzene rings. A range of compounds with interesting biological or photophysical properties contain the phenoxazine core, where the amine moiety is either functionalized or oxidized to the corresponding imine [1–4]. Phenoxazine derivatives can display antitumor activity [5–8], are present in a variety of dyes [9], and can be applied in chemosensors and dye-sensitized solar cells (Figure 1) [10–12]. *N*-Arylphenoxazines were recently employed as photoredox catalysts in metal-free polymerizations [13].

**Scheme 1:**
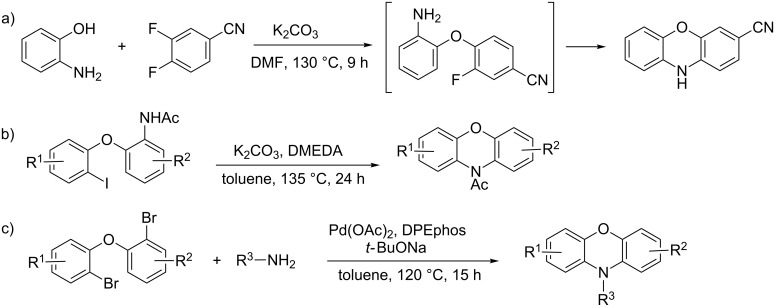
Reported syntheses of phenoxazine derivatives.

The first synthesis of phenoxazine dates back more than 100 years [14], and a range of synthetic routes to this target has since been developed [1]. Transition metal-free routes include the synthesis from 2-aminophenols and 3,4-dihaloarenes decorated with electron-withdrawing substituents, which proceed through a Smiles rearrangement (Scheme 1a) [15–17]. More recently, Bolm and co-workers reported a metal-free cyclization of iodo-substituted diaryl ethers with a broad scope (Scheme 1b) [18]. Transition metal-catalyzed cross couplings have also been employed to form the required C–O and C–N bonds, e.g., by Cu-catalyzed cyclization of 2-(2-bromophenoxy)anilines [17,19–20]. A Pd-catalyzed double *N*-arylation using di(2-bromoaryl) ethers and primary amines was recently developed (Scheme 1c) [21]. Furthermore, *N*-functionalization of the phenoxazine core can be performed under metal-free conditions [22].

Our research group has reported highly efficient *O*-arylations of phenols using diaryliodonium salts [23–25], which are reactive electrophilic arylation reagents [26–28]. As diaryliodonium salts can be easily synthesized in a one-pot manner [28], we envisioned that phenoxazines could be obtained in a straightforward and efficient manner from commercially available starting materials.

**Scheme 2:**
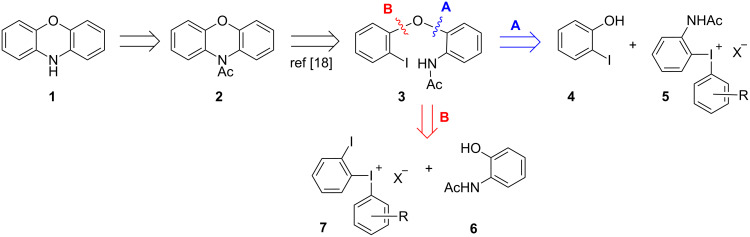
Retrosynthesis of phenoxazine.

According to the retrosynthesis depicted in Scheme 2, target **1** would be formed from acetyl derivative **2**, which can be derived from the functionalized diaryl ether **3** using Bolm’s *N*-arylation (see Scheme 1c) [18]. The synthesis of diaryl ether **3** is the key step of the sequence, proceeding via arylation of phenols with diaryliodonium salts using our reported methodology [23–25]. In reactions with unsymmetrical iodonium salts, this type of *O*-arylation is known to have an *ortho*-effect, i.e., chemoselective transfer of the *ortho*-substituted aryl moiety [29–30]. The use of unsymmetrical iodonium salts facilitates the synthesis of the reagents and avoids waste of an expensive iodoarene, and we hence envisioned chemoselective transfer of the desired aryl moiety from an unsymmetrical salt with a suitable “dummy” aryl group.

Two different approaches to reach **3** are illustrated, either employing 2-iodophenol (**4**) and 2-acetamido-substituted salt **5** (route A) or *N*-functionalized phenol **6** with 2-iodophenyl salt **7** (route B). In route A, the chemoselective transfer of the 2-amido aryl group over the other aryl group would require a quite electron-rich dummy group or the use of a symmetric iodonium salt (R = 2-NHAc). In route B, the challenge would instead be the synthesis of iodonium salt **7**, as the iodo substituent might be prone to oxidation. Furthermore, selective *O*-arylation in the presence of an amide moiety could be challenging, as *N*-arylation of amides with diaryliodonium salts proceeds at room temperature [31–32].

## Results and Discussion

**Scheme 3:**
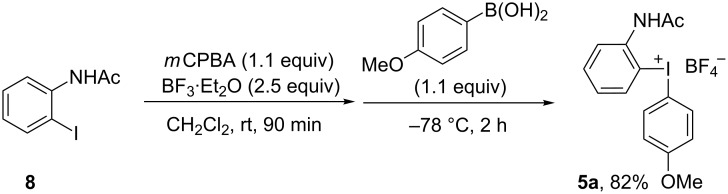
Synthesis of iodonium salt **5a**.

We initially focused on route A and decided to explore an anisyl moiety as dummy group in iodonium salt **5**. The electronic difference between the aryl groups in the formed salt should be sufficient to allow chemoselective transfer of the desired aryl moiety, and the *ortho*-effect exerted by the 2-amido substituent was expected to improve the chemoselectivity further. Salt **5a** was synthesized in good yield using our reported arylboronic acid methodology [33] (Scheme 3). Attempts to form the corresponding tosylate salt, either from iodoarene **8** with anisole and *m*CPBA/TsOH [34] or via anion exchange of **5a** were in vain.

The *O*-arylation of 2-iodophenol (**4**) with salt **5a** was investigated using our previously reported methodology [24–25]. As expected, the reaction proceeded well, both in THF and toluene, of which the latter was most appealing as it might allow a tandem arylation and cyclization to reach *N*-acetylphenoxazine (**2**) in one pot. The arylations were analyzed by crude ^1^H NMR yields using an internal standard and revealed that a minor amount of a byproduct was formed along with the expected diaryl ether **3** (Table 1, entry 1). This was initially believed to be 2-iodophenyl 4-anisyl ether, i.e., the diaryl ether that would form upon transfer of the anisyl moiety to the nucleophile. The reaction was thus further investigated to find conditions that would allow high yield of **3** with complete chemoselectivity.

**Table 1:** Optimization of the *O*-arylation of route A.^a^

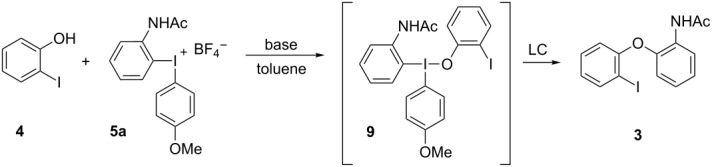							

entry	phenol **4** (equiv)	salt **5a** (equiv)	base (equiv)	*T* (°C)	time (h)	yield **3** (%)^b^	yield byproduct (%)^c^

1	1	1.1	*t-*BuOK (1.1)	rt	18	80	3
2^d^	1	1.1	*t-*BuOK (1.1)	rt	18	67	2
3	1	1.1	NaH (1.1)	rt	18	14	2
4	1	1.1	K_2_CO_3_ (1.1)	rt	18	65	2

5	1	1.5	*t-*BuOK (1.1)	rt	18	77	4
6	1	1.5	*t-*BuOK (1.5)	rt	18	74	14
**7**	**1.5**	**1**	***t-*BuOK (1.5)**	**rt**	**18**	**89 (91)**	**5**

8	1.5	1	*t-*BuOK (1.5)	rt	1	9 (58)	52
9	1	1	*t-*BuOK (1.5)	40	1	49	38
10	1.5	1	*t-*BuOK (1.5)	80	1	79	4
11^e^	1.5	1	*t-*BuOK (1.5)	rt	1	7	trace

^a^Phenol **4** (0.2 mmol) and base were stirred in anhydrous toluene (1 mL) for 30 min at rt before addition of **5a**. The reaction was stirred at the tabulated temperature and time and quenched with brine (see Supporting Information File 1 for details). ^b1^H NMR yield using dibromomethane as internal standard (isolated yield in parenthesis). ^c1^H NMR yield based on the assumption of one OMe group in byproduct, see Supporting Information File 1. ^d^0.5 mL DME as co-solvent. ^e^Quenched with sat. ammonium chloride solution instead of brine.

The amount of byproduct remained approximately constant when solvents and bases were screened (Table 1, entries 1–4, see also Supporting Information File 1), whereas certain changes in stoichiometry influenced the yield (Table 1, entries 5–7). Diaryl ether **3** was efficiently formed using the conditions in entry 7, and could be easily isolated by flash chromatography in 91% yield. A shortening of the reaction time to 1 h profoundly increased the amount of byproduct to 52% (Table 1, entry 8). Surprisingly, the desired product **3** could be isolated in 58% yield from this experiment after prolonged storage of the neat crude product at rt, along with 52% of 4-iodoanisol (see Supporting Information File 1).

This observation is in line with a ligand-coupling (LC) mechanism [30,35–36] where the observed byproduct could be the T-shaped intermediate **9** or the corresponding 4-coordinated species with two phenoxide units [37]. This type of intermediate is expected in LC reactions but can rarely be detected. The groups of Quideau and Muñiz have reported such compounds using an *ortho*-nitro substituted phenol and tetrafluorophthalimide as nucleophiles, respectively [38–39]. Those compounds were isolable at room temperature and could be converted to the arylated products upon heating. The reactivity of **9** is unusual, as it proved stable to neutral work-up with brine, formed product **3** upon longer reaction time or rt storage in CDCl_3_ or neat, but decomposed to starting materials upon work-up with sat. ammonium chloride solution (Table 1, entry 11).

**Scheme 4:**
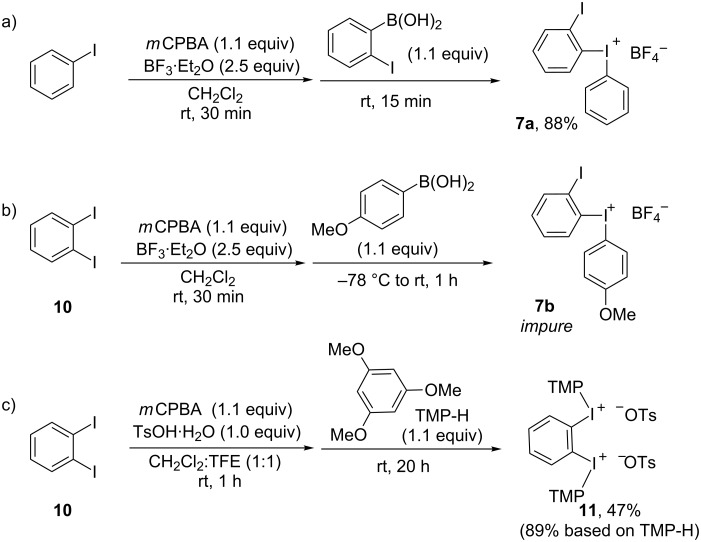
Synthesis of iodonium salt **7**.

Due to the successful outcome of route A, pathway B was only briefly explored. Several dummy groups were considered for the iodonium salt **7**. A phenyl dummy might give sufficient chemoselectivity due to the *ortho*-effect, and the synthesis of salt **7a** was straightforward using 2-iodophenylboronic acid (Scheme 4a). The use of an anisyl dummy proved more difficult, and tetrafluoroborate **7b** was isolated with impurities using the arylboronic acid methodology [33] (Scheme 4b). The *m*CPBA/TsOH methodology [34] with 1,2-diiodobenzene (**10**) and anisole or trimethoxybenzene (TMP-H) as dummy surprisingly delivered the corresponding bisiodonium ditosylates [40–42] instead of the desired **7** (Scheme 4c, see also Supporting Information File 1). The stoichiometry of the reaction did not allow complete formation of **11**, but the yield based on TMP-H was 89%.

**Scheme 5:**
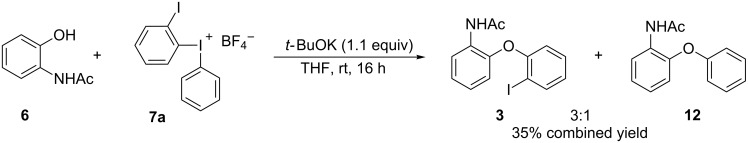
*O*-Arylation via route B.

The arylation of phenol **6** with unsymmetrical diaryliodonium salt **7a** provided the desired diaryl ether **3** in modest yield and limited chemoselectivity (Scheme 5). Competitive *N*-arylation of the amide moiety [31–32] was not observed. Preliminary attempts to employ the bisiodonium salt **11** in the arylation of **6** were fruitless (see Supporting Information File 1), and we hence concluded that route A was most suitable for further investigations.

**Scheme 6:**
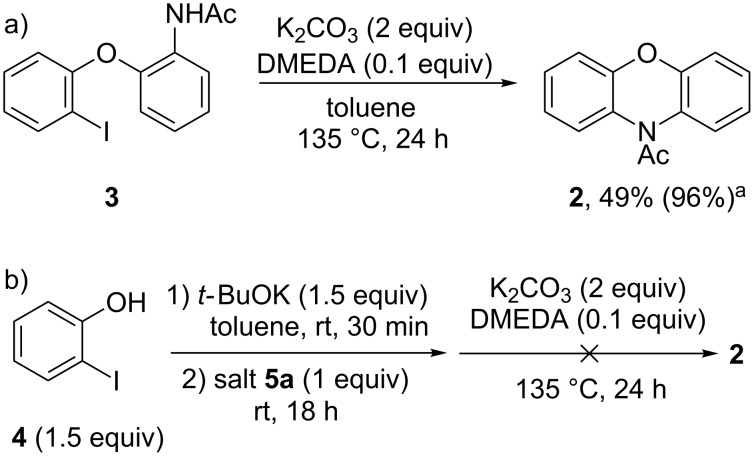
a) Cyclization of diaryl ether **3**. b) Attempted one pot-synthesis of **2**. ^a^Based on recovered **3**.

With high-yielding conditions for the *O*-arylation step at hand (Table 1, entry 7), the literature-reported [18] cyclization of **3** to *N*-acetylphenoxazine (**2**) was performed (Scheme 6a). In our hands, however, product **2** could only be isolated in 49% yield as the cyclization did not reach completion. The ratio of product **2** to starting material **3** remained 1:1 also upon modified conditions (see Supporting Information File 1). Still, the reaction was clean and the yield based on recovered starting material was 96%. The limited conversion into product **2** complicated our aim to perform the arylation and cyclization in one pot. The tandem reaction set up depicted in Scheme 6b delivered the *O*-arylated **3** in 87% yield, rather than product **2**, further illustrating the sensitivity of the cyclization step. Attempts to perform the cyclization with *t-*BuOK and DMEDA only resulted in byproduct formation.

**Scheme 7:**
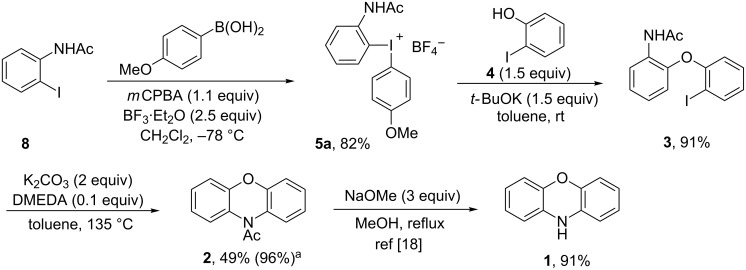
Formal synthesis of phenoxazine (**1**). ^a^Based on recovered **3**.

The deprotection of **2** is reported to proceed in high yield [18], and the synthesis of **2** hence constitutes a metal-free formal synthesis of phenoxazine (**1**). The complete route is depicted in Scheme 7 and delivered *N*-acetylphenoxazine (**2**) in 72% overall yield over 3 steps. Combined with the literature yield for the deprotection, this would provide the target **1** in 65% overall yield based on recovered **3**. The required starting materials in this route can easily be further substituted; hence we expect the methodology to be applicable also for phenoxazine derivatives.

## Conclusion

A transition metal-free formal synthesis of phenoxazine is presented, relying on the *O*-arylation of phenol **4** with unsymmetrical diaryliodonium salt **5a** to provide substituted diaryl ether **3** as the key step. This species underwent an intramolecular *N*-arylation to provide the cyclized product **2**. The overall yield in this three-step sequence is 72% based on recovered diaryl ether. Interestingly, an unusually stable iodine(III) intermediate was formed in the *O*-arylation. This species survived neutral work-up and could be converted to product upon longer reaction time.

## Supporting Information

File 1 Experimental details for the synthesis of starting materials and products, analytical data for products **2**, **3**, **5a**, **7a, 9** and **11**.

File 2 NMR spectra for products **2**, **3**, **5a**, **7a** and **11**.
