# Supplementary material for: Metal-free formal synthesis of phenoxazine
Source: Beilstein J Org Chem. 2018 Jun 20;14:1491–7. doi: 10.3762/bjoc.14.126 (PMC6036965; doi:10.3762/bjoc.14.126)
Supplement: File 1 — Experimental details for the synthesis of starting materials and products, analytical data for products 2, 3, 5a, 7a, 9 and 11. [file Beilstein_J_Org_Chem-14-1491-s001.pdf]

Supporting Information

for

Metal-free formal synthesis of phenoxazine

Gabriella Kervefors, Antonia Becker, Chandan Dey and Berit Olofsson*

Address: Department of Organic Chemistry, Arrhenius Laboratory, Stockholm University, SE-106-91 Stockholm, Sweden

* Corresponding author

Email: Berit Olofsson - berit.olofsson@su.se

Experimental details for the synthesis of starting materials and products, analytical data for products 2, 3, 5a, 7a, 9 and 11

Table of contents

1 GENERAL EXPERIMENTAL PROCEDURE S2

2 ACETYLATION OF ANILINES S2

3 SYNTHESIS OF DIARYLIODONIUM SALTS S3

4 *O*-ARYLATION OF 2-IODOPHENOL VIA ROUTE A S5

4.1 OPTIMIZATION AND BYPRODUCT FORMATION S5
4.2 YIELD DETERMINATION USING INTERNAL STANDARD S6
4.3 STRUCTURE ELUCIDATION S7
4.4 SYNTHESIS OF *N*-(2-(2-IODOPHENOXY)PHENYL)ACETAMIDE (3) S9

5 *O*-ARYLATION OF PHENOL 6 VIA ROUTE B S10

6 CYCLIZATION AND ONE-POT REACTIONS S12

6.1 CYCLIZATION OF ETHER 3 ..... S12
6.2 ATTEMPTED ONE-POT SYNTHESIS OF *N*-ACETYLPHENOXAZINE (2) S13

7 REFERENCES S13

1 General experimental procedure

All arylations were carried out in oven-dried glassware unless otherwise noted. Toluene was dried using a VAC-purification system. Boron trifluoride etherate and DMEDA were either newly purchased or distilled prior to use. *m*CPBA (Aldrich, 77% active oxidant) was dried at rt on high vacuum for several hours, and titrated by iodometric titration [1] prior to use. TLC analysis was performed on pre-coated Merck silica gel 60 F254 plates using UV light. Flash column chromatography was done on SiO_2 purchased from Aldrich (technical grade, 60 Å pore size, 230–400 mesh, 40–63 µm). Melting points were measured using a STUART SMP3 and are reported uncorrected. The melting point measurements refer to the solidified materials as the result of the given experimental procedures, no additional recrystallization was done. All NMR spectra were recorded using a 400 MHz Bruker AVANCE II with a BBO probe at 298 K using $CDCl_3$, MeOD-d_4 or DMSO-d_6 as solvent. Chemical shifts are given in ppm relative to the residual solvent peak (^{1}H NMR: $CDCl_3$ δ 7.26; MeOD-d_4 δ 3.31; DMSO-d_6 δ 2.50; ^{13}C NMR: $CDCl_3$ δ 77.16; MeOD-d_4 δ 49.00; DMSO-d_6 δ 39.52) with multiplicity (br = broad, s = singlet, d = doublet, t = triplet, m = multiplet, app = apparent), coupling constants (in Hz) and integration. High-resolution mass analyses were obtained using a Bruker microTOF ESI. Full analytical data is given for compounds that are novel or not fully characterized in the literature; ^{1}H NMR and ^{13}C NMR are given for literature reported compounds.

2 Acetylation of anilines

N-(2-Hydroxyphenyl)acetamide (6)

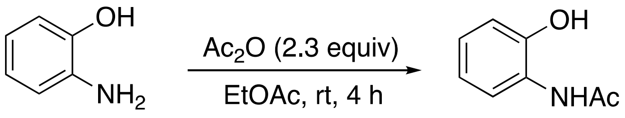

6 97%

2-Aminophenol (1.9 g, 16.9 mmol, 1.0 equiv) was dissolved in ethyl acetate (25 mL) and acetic anhydride (3.7 mL, 39.1 mmol, 2.3 equiv) was added dropwise at 0 °C. The reaction mixture was stirred at room temperature for 4 h and a light yellow solid precipitated, then concentrated under reduced pressure, leaving approximately 5 mL of solvent. Pentane was added and the mixture stirred for an additional 30 min. The mixture was filtered and the light brown solid was washed with pentane and dried under reduced pressure to give amide **6** (2.478 g, 16.39 mmol, 97%). The analytical data are consistent with previous reports [2].

^{1}H NMR (400 MHz, MeOD-d_4) δ 7.57 (dd, *J* = 8.0, 1.6 Hz, 1H), 6.99 (ddd, *J* = 8.0, 7.4, 1.6 Hz, 1H), 6.88 – 6.73 (m, 2H), 2.17 (s, 3H). ^{13}C NMR (101 MHz, MeOD-d_4) δ 172.2, 149.7, 127.1, 126.8, 123.9, 120.6, 117.3, 23.4.

N-(2-Iodophenyl)acetamide (8)

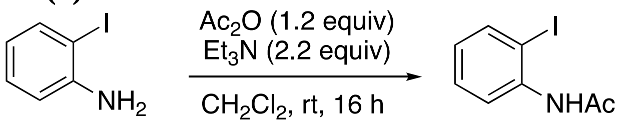

8 75%

2-Iodoaniline (2.2 g, 10.1 mmol, 1.0 equiv) and triethylamine (3.1 mL, 22.2 mmol, 2.2 equiv) were dissolved in CH_2Cl_2 (25 mL) under argon atmosphere at rt, followed by dropwise addition of acetic anhydride (1.2 mL, 12.1 mmol, 1.2 equiv) at 0 °C. The mixture stirred over night at room temperature to completion after 19 h, as judged by TLC. The reaction was quenched with water and extracted with CH_2Cl_2. The combined organic phases were washed with saturated solutions of sodium bicarbonate and ammonium chloride, dried over anhydrous magnesium sulfate and concentrated under vacuum. The crude product was purified by column chromatography

(pentane:EtOAc 4:1) to give amide **8** (1.987 g, 7.61 mmol, 75%) as colorless crystals. The analytical data are consistent with previous reports [3].
^{1}H-NMR (400 MHz, $CDCl_3$): δ 8.21 (d, *J* = 8.2 Hz, 1 H), 7.78 (d, *J* = 7.8 Hz, 1 H), 7.41 (br s, 1 H), 7.35 (td, *J* = 7.8, 1.3 Hz, 1 H), 6.85 (app t, *J* = 7.7 Hz, 1 H), 2.24 (s, 3 H). ^{13}C NMR (101 MHz, $CDCl_3$) δ 168.3, 138.8, 138.3, 129.3, 126.1, 122.4, 90.3, 24.8.

3 Synthesis of diaryliodonium salts

2-Acetamidophenyl(4-methoxyphenyl)iodonium tetrafluoroborate (5a)

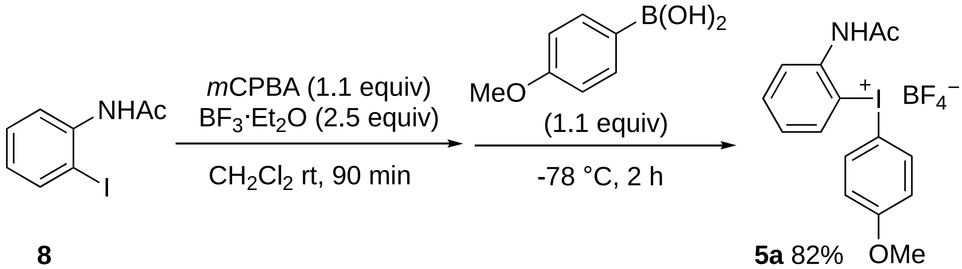

8 **5a** 82%

Amide **8** (0.78 g, 3.0 mmol, 1.0 equiv) and *m*CPBA (0.66 g, 3.3 mmol, 1.1 equiv) were dissolved in CH_2Cl_2 (10 mL) and stirred at 0 °C for 15 min. Boron trifluoride etherate (0.95 mL, 7.5 mmol, 2.5 equiv) was added dropwise at 0 °C, then the reaction mixture was stirred at room temperature for 90 min. A solution of (4-methoxyphenyl)boronic acid (0.50 g, 3.3 mmol, 1.1 equiv) in CH_2Cl_2 (3 mL) was added dropwise at −78 °C. The reaction was stirred at −78 °C for 2 h and an additional 30 min at room temperature followed by filtration through a silica plug (50 mL CH_2Cl_2, then 250 mLCH_2Cl_2:MeOH 20:1). The latter solution was concentrated under reduced pressure at room temperature, giving the crude as an orange sticky solid. Diethyl ether (50 mL) was added to the crude followed by stirring rapidly for 1 h until a white precipitation occurred. The mixture was stored in the freezer overnight. The ether layer was then decanted off and the residual substance was stirred again with diethyl ether (50 mL) and stored in the freezer for 30 min. The ether layer was decanted off and the remaining solid was washed with pentane by decantation three times. The residue was dried under high vacuum to give **5a** (1.117 g, 2.46 mmol, 82%) as a beige solid.
^{1}H NMR (400 MHz, $CDCl_3$) δ 9.90 (s, 1H), 7.92 (d, *J* = 9.0 Hz, 2H), 7.57-7.51 (m, 2H), 7.41 – 7.35 (m, 1H), 7.15-7.11 (m, 1H), 7.01 (d, *J* = 9.0 Hz, 2H), 3.87 (s, 3H), 2.35 (s, 3H). ^{13}C NMR (101 MHz, $CDCl_3$) δ 174.9, 163.9, 138.7, 137.8, 133.4, 132.0, 129.3, 127.0, 118.6, 110.0, 102.3, 56.0, 23.5. ^{19}F NMR (377 MHz, $CDCl_3$) δ -149.6, -149.6. Mp = 116.5–118.9 °C. HRMS (ESI): calcd for $C_{15}H_{15}INO_2$ $[M-BF_4]^+$: 368.9142; found: 368.9138.

2-Iodophenyl(phenyl)iodonium tetrafluoroborate (7a)

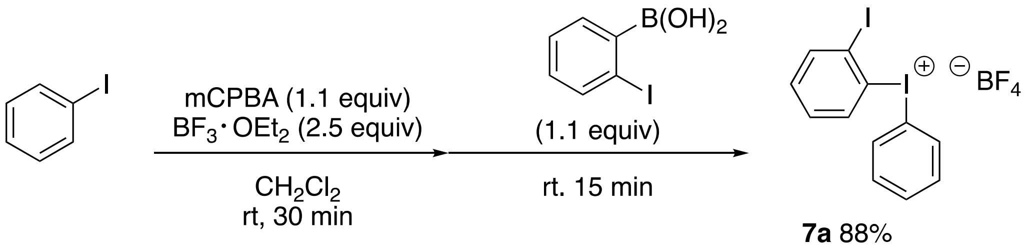

7a 88%

*m*CPBA (1.4 mmol, 1.1 equiv) was dissolved in CH_2Cl_2 (6 mL) followed by the addition of iodobenzene (0.14 mL, 1.3 mmol, 1.0 equiv) and BF_3·OEt_2 (0.4 mL, 3.2 mmol, 2.5 equiv) at 0 °C. The resulting yellow solution was stirred at rt for 30 min and then cooled to 0 °C, and (2-iodophenyl)boronic acid (0.35 g, 1.4 mmol, 1.1 equiv) was added. After 15 min of stirring at rt, the crude was applied on a silica plug (0.8 g) and eluted with CH_2Cl_2 (50 mL), followed by CH_2Cl_2:MeOH (140 mL, 20:1). The latter solution was concentrated, and diethyl ether (15 mL) was added to the residue to induce a precipitation of salt **7a**. The mixture was stored in the freezer for 30

min. The ether layer was then decanted off and the residual substance was stirred again with diethyl ether (50 mL) and stored in the freezer for 30 min. The ether layer was again decanted off and the remaining residue was dried under high vacuum to give **7a** (0.563 g, 1.14 mmol, 88%) as a white solid.
^{1}H NMR (400 MHz, DMSO-d_6) δ 8.52-8.50 (m, 1H), 8.17-8.12 (m, 3H), 7.68-7.65 (m, 1H), 7.59 – 7.47 (m, 3H), 7.41-7.37 (m, 1H). ^{13}C NMR (101 MHz, DMSO-d_6) δ 140.1, 138.4, 134.6, 133.7, 131.9, 131.8, 131.0, 129.8, 118.1, 105.8. ^{19}F NMR (377 MHz, DMSO-d_6) δ -148.2, -148.3. Mp = 148.4 – 152.8 °C (decomposed). HRMS (ESI): calcd for $C_{12}H_9I_2$ $[M-BF_4]^+$: 406.8788; found: 406.8790.

2-Iodophenyl(4-methoxyphenyl)iodonium tetrafluoroborate (7b)

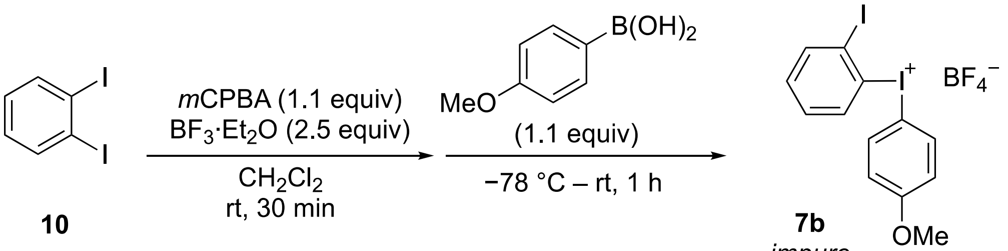

10 **7b** *impure*

*m*CPBA (3.3 mmol, 1.1 equiv) was dissolved in CH_2Cl_2 (10 mL) followed by the addition of 1,2-diiodobenzene (**10**, 0.39 mL, 3.0 mmol, 1.0 equiv) and $BF_3 \cdot OEt_2$ (0.95 mL, 7.5 mmol, 2.5 equiv) at 0 °C. The resulting yellow solution was stirred at rt for 30 min and then cooled to 0 °C, and (4-methoxyphenyl)boronic acid (0.51 g, 3.33 mmol, 1.1 equiv) in DCM (2 mL) was added dropwise at −78 °C. The mixture was stirred at −78 °C for 30 min followed by 60 min at rt. The mixture was applied on a silica plug and eluted with CH_2Cl_2 (130 mL), followed by CH_2Cl_2:MeOH (20:1, 300 mL). The latter solution was concentrated, and diethyl ether (15 mL) was added to the residue to induce a precipitation of salt **7b**. The mixture was stirred at rt for 1 h and then stored in the freezer overnight. The white solid was filtered off, washed with diethyl ether (10 mL × 4) and the remaining residue was dried under high vacuum to give **7b** (0.563 g, 1.14 mmol) as a white solid. ^{1}H NMR analysis showed that impurities remained.

1,2-Phenylene-bis(2,4,6-trimethoxyphenyliodonium) ditosylate (11)

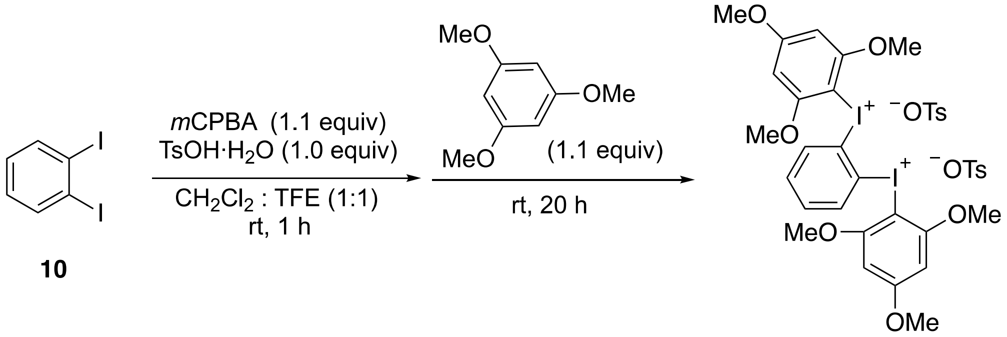

10

11 47% based on **10** (89% based on TMP-H)

*m*CPBA (3.3 mmol, 1.1 equiv) was dissolved in CH_2Cl_2 (6 mL) and TFE (6 mL) followed by the addition of 1,2-diiodobenzene (**10**) (0.39 mL, 3.0 mmol, 1.0 equiv) and $TsOH \cdot H_2O$ (0.57 g, 3.0 mmol, 1.0 equiv) at room temperature. The resulting yellow solution was stirred at rt for 1 h and then cooled to 0 °C, and 1,3,5-trimethoxybenzene (TMP-H) (0.56 g, 3.3 mmol, 1.1 equiv) dissolved in CH_2Cl_2 (2 mL) was added. The reaction was stirred overnight at rt. The crude was concentrated and diethyl ether (30 mL) was added to the residue to induce a precipitation of salt **11**. The mixture was stored in the freezer overnight. The white solid was filtered off, washed with diethyl ether (10 mL × 4) and the remaining residue was dried under high vacuum to give **11** (1.47 g, 1.46 mmol, 89%) as a white solid. The compound was analyzed by both 1D and 2D NMR techniques, the HSQC spectrum is attached together with the ^{1}H NMR and ^{13}C NMR at the end.

^{1}H NMR (400 MHz, DMSO-d_6) δ 8.35-8.31 (m, 2H), 7.70-7.68 (m, 2H), 7.45 (d, J = 8.0 Hz, 4H), 7.10 (d, J = 8.0 Hz, 4H), 6.50 (s, 4H), 3.96 (s, 12H), 3.88 (s, 6H), 2.29 (s, 6H). ^{13}C NMR (101 MHz, DMSO) δ 166.6, 159.8, 145.7, 139.5, 137.6, 134.1, 128.0, 125.5, 123.1, 92.2, 87.3, 57.2, 56.3, 20.8. Mp = 193.9–195.1 °C.

4 *O*-Arylation of 2-iodophenol via route A

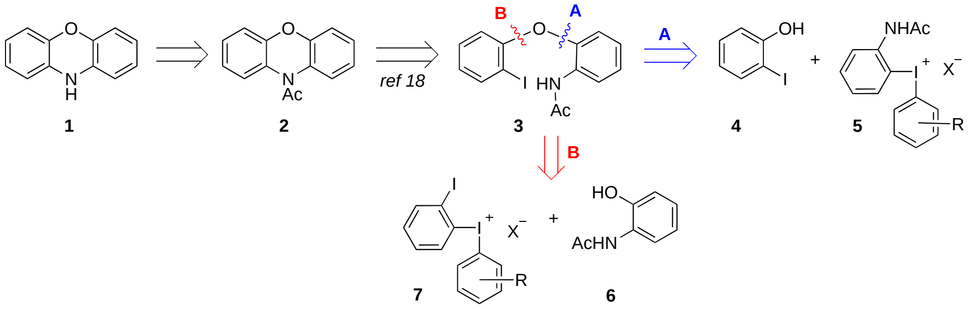

Scheme S1: Retrosynthesis.

4.1 Optimization and byproduct formation

The optimization results are given in Table S1, using the following general procedure:
2-Iodophenol (**4**, 0.2 mmol) and base were dissolved in dry solvent (1 mL) in an oven-dried microwave vial and stirred at room temperature for 30 min. Salt **5a** was added to the solution, in tabulated entries followed by DME (0.5 mL). The vial was capped and the reaction mixture was stirred at the given temperature for the given time. The reaction mixture was quenched with brine (10 mL) and the aqueous phase was extracted three times with EtOAc (10 mL). The combined organic phases were dried with anhydrous sodium sulfate and the solution was concentrated under vacuum to give the crude product. Yields were determined as described in Section 4.2.

Table S1. Optimization of route A.

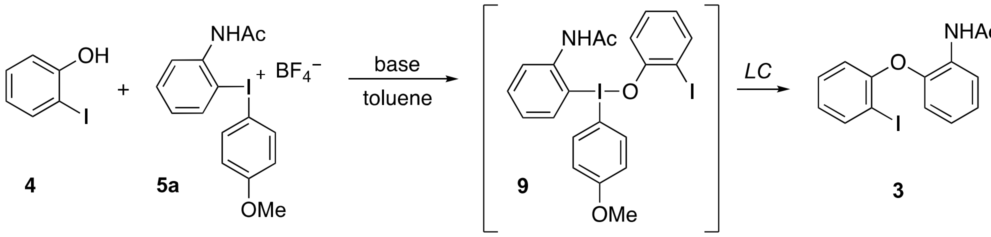

Entry	Phenol **4** (equiv)	Salt **5a** (equiv)	Solvent	Base (equiv)	Temp (°C)	Time (h)	Yield **3** (%)[a]	Yield of by-product (%)[a]
1	1	1.1	toluene	NaH (1.1)	rt	18	14	2
2	1	1.1	toluene	K_2CO_3 (1.1)	rt	18	65	2
3	1	1.5	toluene	K_2CO_3 (1.1)	rt	18	11	1
4	1	1.5	toluene	K_2CO_3 (1.5)	rt	18	25	4
5	1	1.1	toluene	*t*-BuOK (1.1)	rt	18	80	3
6[b]	1	1.1	toluene	*t*-BuOK (1.1)	rt	18	67	2
7	1	1.1	THF	*t*-BuOK (1.1)	rt	18	45	28
8[b]	1	1.1	THF	*t*-BuOK (1.1)	rt	18	35	19
9[b]	1	1.1	THF	*t*-BuOK (1.1)	rt	19	37	11
10	1	1.1	THF	*t*-BuOK (1.1)	rt	16	(87)	
11[c]	1	1	THF	*t*-BuOK (1.1)	rt	16	(88)	
12	1	1.5	toluene	*t*-BuOK (1.1)	rt	18	77	4

13	1	1.5	toluene	*t*-BuOK (1.5)	*rt*	18	74	14
14	1.5	1	toluene	*t*-BuOK (1.5)	rt	18	89 (91)	5
15[d]	1.5	1	toluene	t-BuOK (1.5)	rt	18	(89)	
16	1.5	1	toluene	*t*-BuOK (1.5)	rt	1	9 (58)[e]	52
17	1.5	1	toluene	*t*-BuOK (1.5)	rt	1	(11)	
18[f]	1.5	1	toluene	*t*-BuOK (1.5)	rt	1	7	-
19	1	1	toluene	*t*-BuOK (1.1)	40	1	49	38
20	1.5	1	toluene	*t*-BuOK (1.5)	80	1	79	4
21	1.5	1	toluene	*t*-BuOK (1.5)	rt	4	57	19

[a] ^{1}H NMR-yield using dibromomethane as internal standard (isolated yield in parenthesis). [b] DME added as co-solvent. [c] 1.5 mL solvent used. [d] 0.6 mmol scale. [e] Concentrated crude stored open to air over 48 h before isolation by column. [f] Quenched with sat. ammonium chloride solution instead of brine.

4.2 Yield determination using internal standard

Dibromomethane (IS, 2 equiv) was added to the crude product, the mixture was dissolved in $CDCl_3$, and the yields were calculated from the crude ^{1}H NMR spectrum using the following peaks: δ IS 4.93 (s, 2H x2); product **3** 8.43 (dd, 1H) & 2.20 (s, 3H); byproduct: 3.82 (s) and 2.31 (s). These peaks were assumed to be 3H, originating from OMe and NAc, respectively. The peaks at 2.2–2.3 had better baseline separation, and were hence used for integrations.

Figure S1 shows the crude ^{1}H NMR (400 MHz) of the experiment in Table S1, entry 7. In S1**A**, the integration of the IS is set to 4 (2H x2). In S1**B**, the IS integration is adjusted to directly show the yield of product **3** (45%) and byproduct (28%).

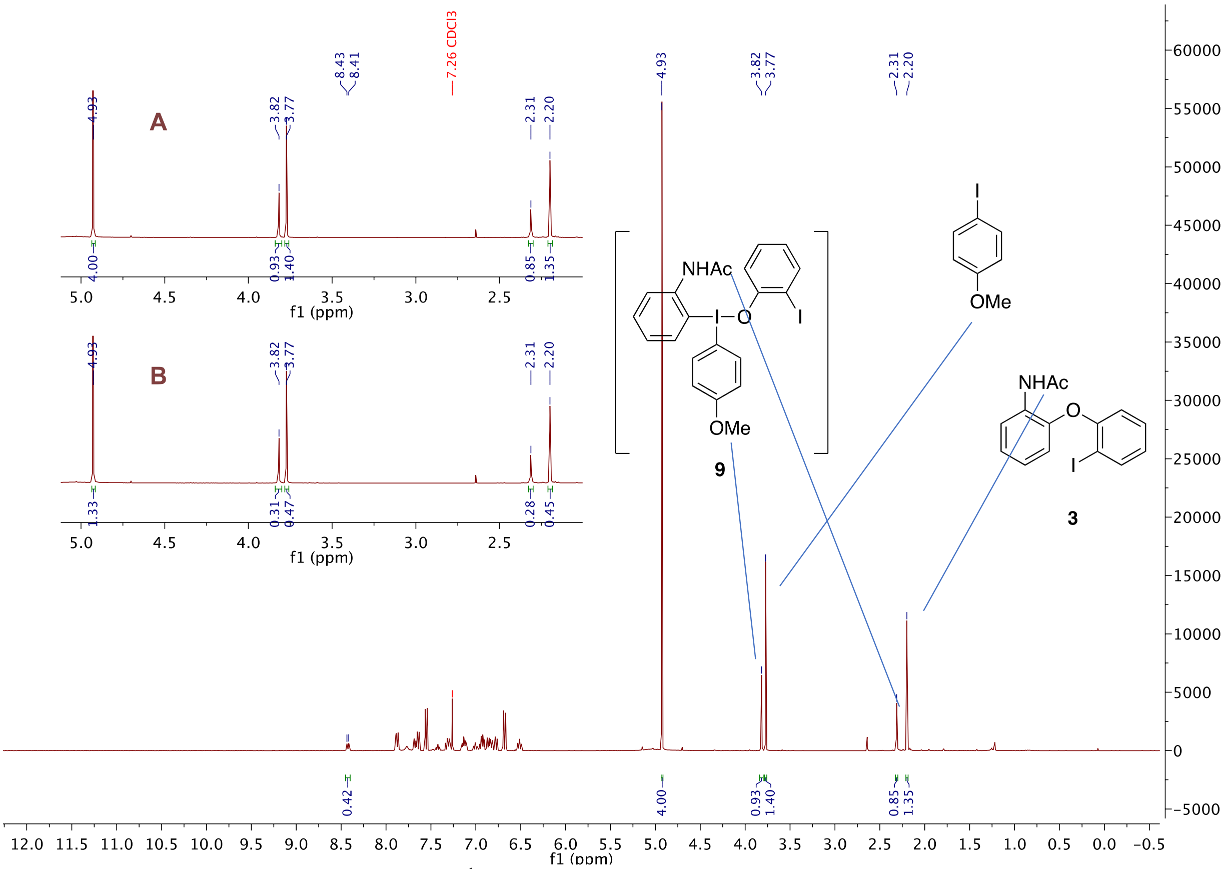

Figure S1. Calculation of ^{1}H-NMR yield from CH_2Br_2 as internal standard.

4.3 Structure elucidation

We believe that the byproduct described in the manuscript and this SI section is a reaction intermediate that is stable enough to survive standard workup conditions followed by ^{1}H NMR analysis of the crude reaction mixture. We suggested the structure of this intermediate to be **9**, due to experimental observations and preliminary NMR studies. The intermediate slowly converted into diaryl ether **3** under various conditions:

1) Prolonged reaction time at rt (compare entries 14, 16, 21) or upon heating (entries 19-20).
2) Storage of the crude reaction (after workup) in $CDCl_3$ – the ratio of **3**:**9** slowly increased towards **3** upon several sequential NMR experiments of the same sample
3) Storage of the crude reaction (after workup) without solvent.

Compound **9** could not be isolated in pure form, as diaryl ether **3** and iodoanisole, generated upon ligand coupling of **9**, formed at the same reaction temperature and **9** did not survive column chromatography.

Figure S2 shows a comparison of ^{1}H NMR spectrum of the crude reaction, after workup with brine, to the spectra of the starting materials (phenol **4** and iodonium salt **5a)** and products (diaryl ether **3** and iodoanisole). The crude spectrum shows that no phenol or salt remains in the mixture. Integrations show that the diaryl ether **3** and the iodoarene are formed in a 1:1 ratio (see Figure S3). The singlets generated from the OMe and NAc groups, originating from **5a**, have shifted slightly. This indicates that the T-shaped intermediate **9** has formed. Quideau and coworkers have successfully isolated such an iodine(III) compound using a nitrophenol as nucleophile, and observed an up-field shift in the NMR when comparing the intermediate to the starting material [4]. The same trend is observed in the NMR from the *O*-arylation of 2-iodophenol **4**. There are newly formed peaks shifted up-field in the crude NMR that share the same coupling pattern as the phenol **4**. Some peaks interfere with signals from the diaryl ether **3**, making the integration difficult.

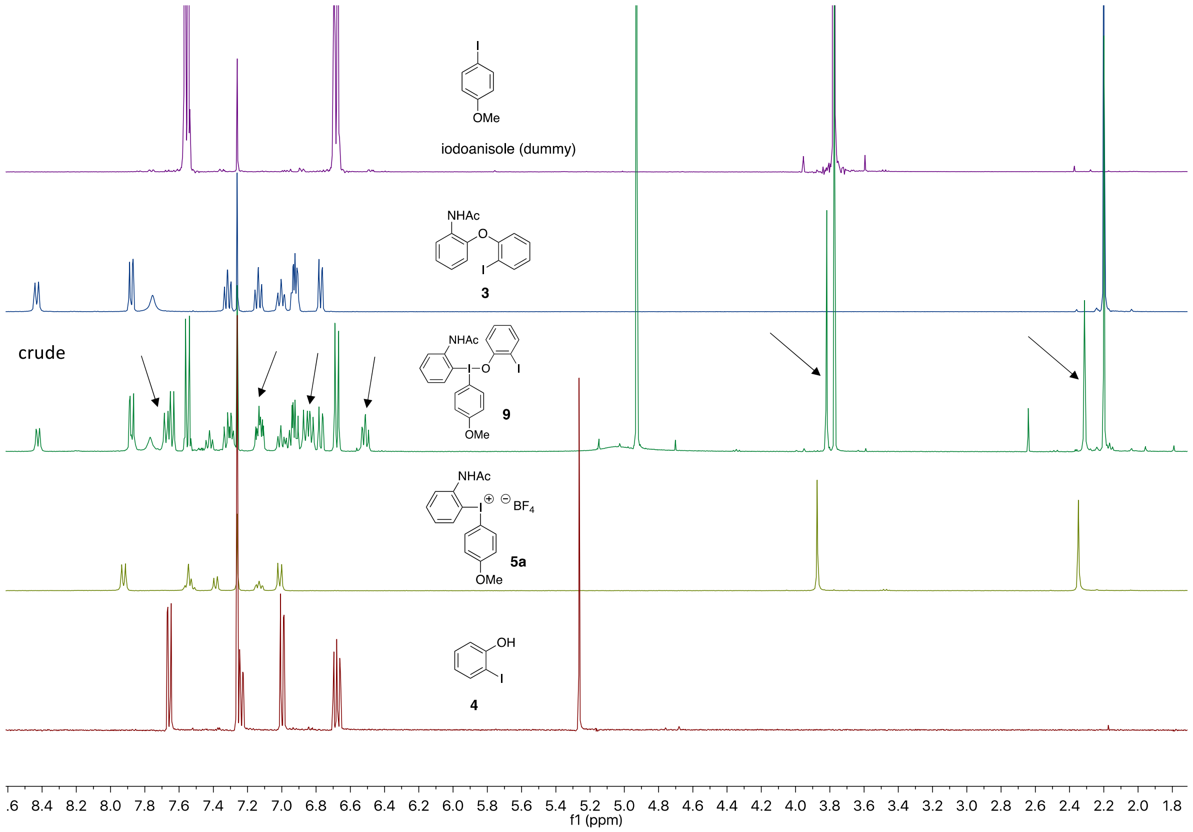

Figure S2. NMR (400 MHz, $CDCl_3$) comparison of starting materials, crude reaction (Table S1, entry 7), product and formed iodoarene.

Structure determination of **9** remains difficult despite a range of NMR experiments, both due to the mixture of compounds in the spectrum, and because the ratio of **3**:**9** varies within long NMR experiments. The crude reaction mixture in $CDCl_3$ hence seems to be a dynamic system where several possible complexes can form, as depicted in Figure S3.

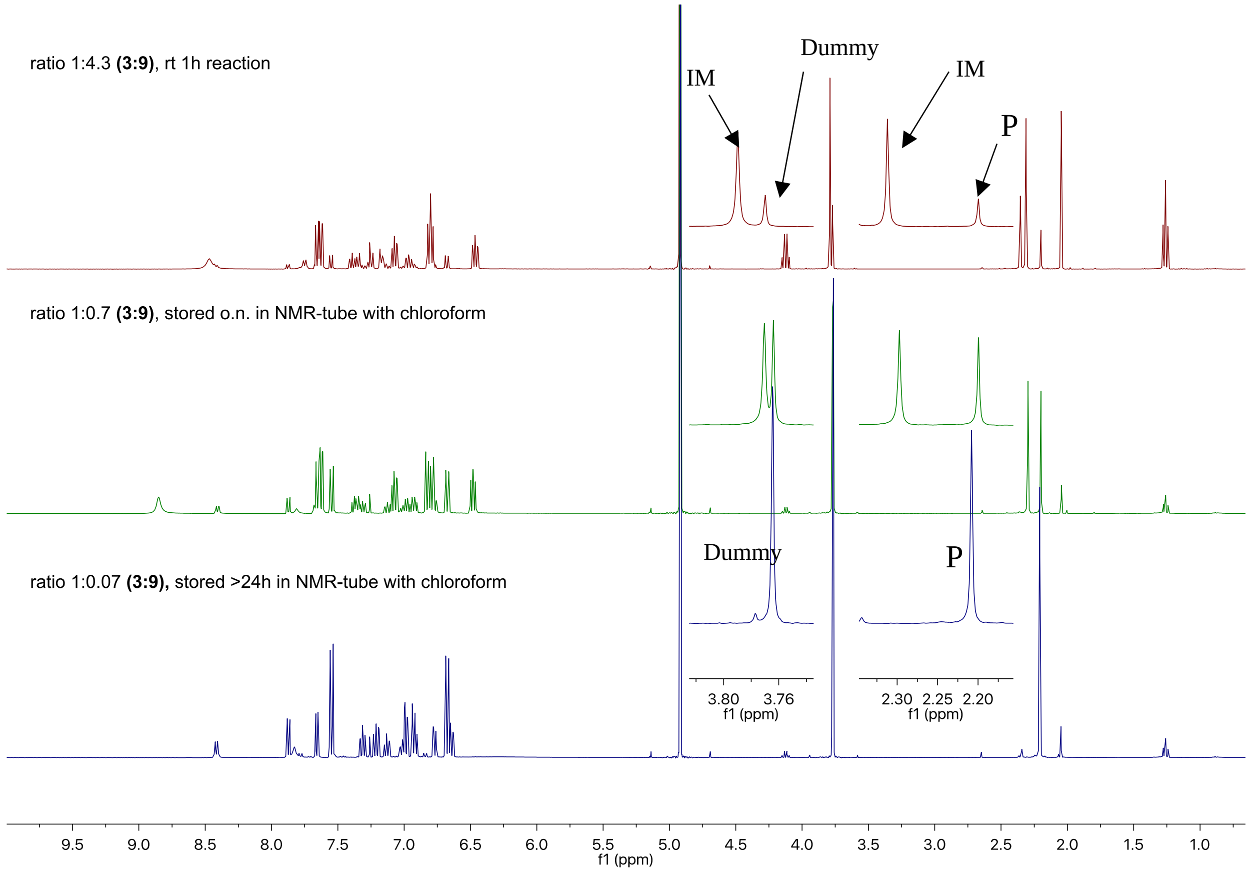

Figure S3. Formation of ether **3** after 1 h reaction time, at room temperature, and the conversion of the intermediate over time upon storage in the NMR tube ($CDCl_3$ medium).

Figure S4 shows the ^{1}H NMR of a reaction with a **3**:**9** ratio of 14:57 according to integration of the NAc peaks. Some signals from the product interfere with those of the intermediate, causing problems in the integration. Integration values are given by recalculating the values based on the known amount of product in each peak (assinged as * in Figure S4). The suggested structure **9** is color coded and the peaks thatwe believe belong to each aryl groups are indicated in the spectrum. The assignement is based on COSY spectra from several different ratios of **3**:**9**, one of which is found in Supporting Information File 2. Unfortunately, NOESY spectra did not provide any additional information.

In this reaction, the integrals match very well with an intermediate structure that contains two phenoxy groups, and the structure could very well be 4-coordinated **9’** rather than 3-coordinated **9**. We have recently reported a mechanistic study where certain O-arylations were suggested to proceed through 4-coordinated intermediates similar to **9’** [5]. The reaction employs 1.5 equiv of phenol **4**, so formation of **9’** would indeed be possible based on stoichiometry and the observed amount of intermediate (approximately 50% at most). Since the individual integrations of the peaks vary somewhat between reactions, further studies would be necessary to determine whether the main intermediate is **9** or **9’**, or whether both exist in fast equilibrium.

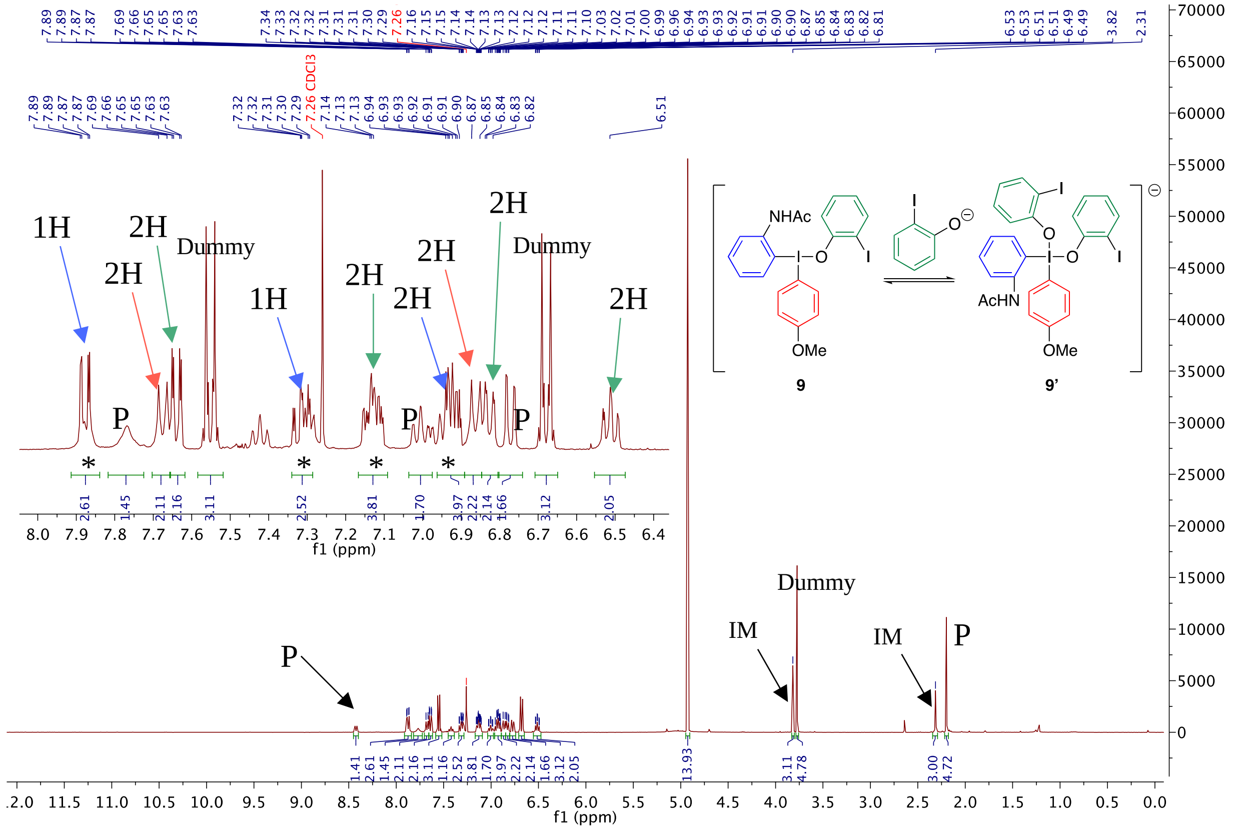

Figure S4. ^{1}H MR (400 MHz, $CDCl_3$) of crude reaction containing intermediate (IM), product and dummy.

(2-Acetamidophenyl)(2-iodophenoxy)(4-methoxyphenyl)-λ[3]-iodane (9) or bis(2-iodophenoxy) species 9'. ^{1}H NMR (400 MHz, $CDCl_3$) δ 7.88 (dd, *J* = 8.2, 1.6 Hz, 1H), 7.68 (d, *J* = 8.7 Hz, 2H), 7.64 (dd, *J* = 7.9, 1.5 Hz, 2H), 7.35 – 7.29 (m, 1H), 7.15 – 7.10 (m, 2H), 6.96 – 6.89 (m, 2H), 6.86 (d, *J* = 8.7 Hz, 2H), 6.83 (dd, *J* = 8.1, 1.5 Hz, 2H), 6.53 – 6.49 (m, 2H), 3.82 (s, 3H), 2.31 (s, 3H).

4.4 Synthesis of *N*-(2-(2-Iodophenoxy)phenyl)acetamide (3)

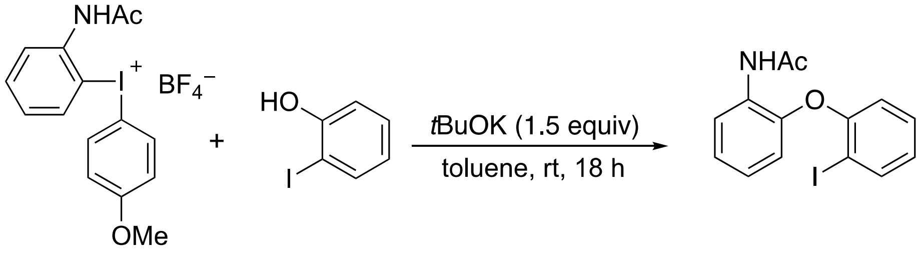

5a (1 equiv) **4** (1.5 equiv) **3** 91%

2-Iodophenol (**4**) (0.066 g, 0.30 mmol, 1.5 equiv) and *t*-BuOK (0.034 g, 0.30 mmol, 1.5 equiv) were dissolved in anhydrous toluene (1 mL) in an oven-dried microwave vial and stirred at room temperature for 30 min. Salt **5a** (0.091 g, 0.20 mmol, 1 equiv) was added and the vial was capped. The dark brown reaction mixture was stirred at room temperature for 18 h and then quenched with brine (10 mL) and the aqueous phase was extracted with EtOAc (30 mL × 3). The organic phases were dried over anhydrous sodium sulfate and concentrated under vacuum. The crude product was purified by column chromatography (pentane:EtOAc, 10:1 → 4:1) to give diaryl ether **3** (0.064 g, 0.18 mmol, 91%) as beige/off white solid. The analytical data are consistent with previous reports apart from small differences in the ^{1}H NMR spectrum [6].

R_f = 0.27 in pentane:EtOAc 4:1. ^{1}H NMR (400 MHz, $CDCl_3$) δ 8.43 (d, *J* = 8.2, 1H), 7.88 (dd, *J* = 8.2, 1.6 Hz, 1H), 7.75 (br s, 1H), 7.32 (td, *J* = 7.9, 1.5 Hz, 1H), 7.14 (td, *J* = 7.9, 1.5 Hz, 1H), 7.01

(td, J = 7.8, 1.6 Hz, 1H), 6.95 – 6.87 (m, 2H), 6.77 (dd, J = 8.2, 1.6 Hz, 1H), 2.20 (s, 3H). ^{13}C NMR (101 MHz, $CDCl_3$) δ 168.5, 155.6, 145.1, 140.1, 130.1, 129.6, 126.1, 124.6, 124.0, 121.3, 119.2, 117.4, 88.6, 25.1. Mp = 98.1–100.9 °C. HRMS (ESI): calcd for $C_{14}H_{12}INaNO_2$ $[M+Na]^+$: 375.9805; found: 375.9802.

5 *O*-Arylation of phenol 6 via route B

N-(2-(2-Iodophenoxy)phenyl)acetamide (3)

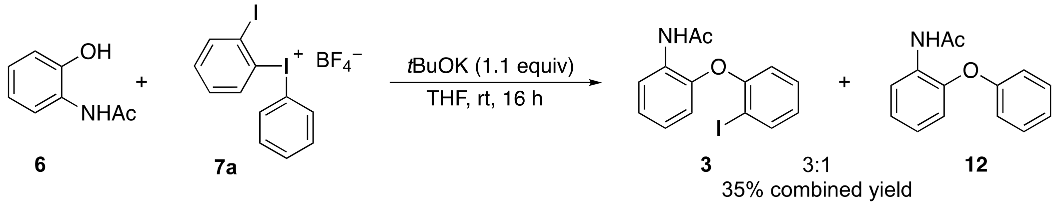

Amide **6** (0.030 g, 0.2 mmol, 1.0 equiv) and *t*-BuOK (0.025 g, 0.22 mmol, 1.1 equiv) were dissolved in anhydrous THF (1 mL) at 0 °C and then allowed to reach room temperature and stirred for 15 min. Salt **7a** (0.099 g, 0.2 mmol, 1 equiv) dissolved in THF (0.5 mL) was added, the vial was capped and the mixture was stirred at room temperature for 18 h. The reaction was quenched with brine (10 mL) and the aqueous phase was extracted with EtOAc (30 mL × 3). The organic phases were dried over anhydrous sodium sulfate and concentrated under vacuum. The crude product was purified by column chromatography (pentane:EtOAc 3:1 to give an inseparable mixture of amide **3** and *N*-(2-phenoxyphenyl)acetamide **12** (0.023 g, ratio 3:1, 35% combined yield). See Section 4.3 for complete analytical data of ether **3**.

Integrated peaks to determine the product ratio are depicted in Figure S5:

Product **3:** ^{1}H NMR (400 MHz, $CDCl_3$) δ 6.77 (dd, J = 8.2, 1.6 Hz, 1H), 2.20 (s, 3H).

Product **12:** ^{1}H NMR (400 MHz, $CDCl_3$) δ 6.84 (dd, J = 8.1, 1.3 Hz, 1H), 2.17 (s, 3H).

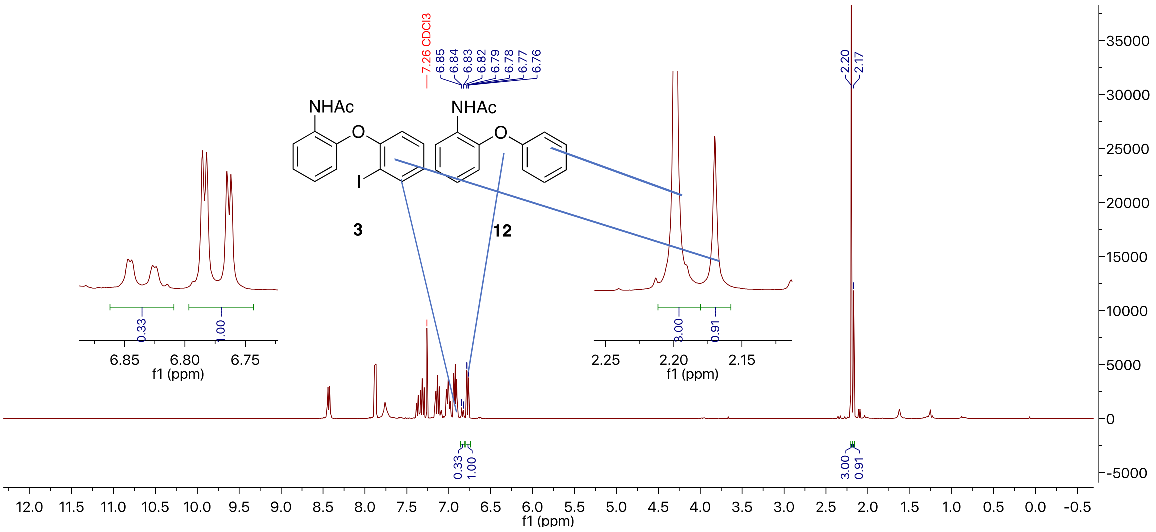

Figure S5. NMR of inseparable mixture of diaryl ethers **3** and **12**.

Attempted arylation with bisiodonium salt 11

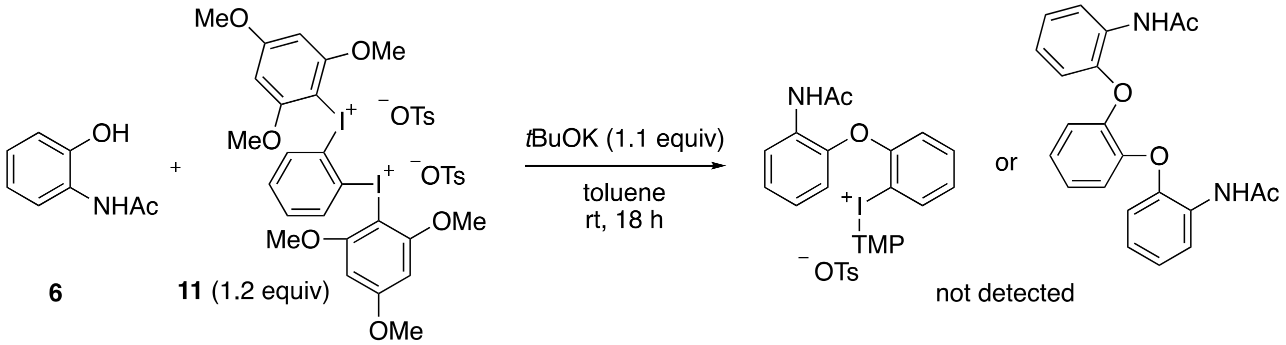

Amide **6** (0.015g, 0.1 mmol, 1.0 equiv) and *t*-BuOK (0.012 g, 0.11 mmol, 1.1 equiv) were dissolved in anhydrous toluene (1 mL) and stirred at room temperature for 30 min. Salt **11** (0.12 g, 0.12 mmol, 1.2 equiv) was added and the vial was capped. The dark brown reaction mixture was stirred at room temperature for 18 h. The solvent was removed under reduced pressure and the crude was analyzed by ^{1}H NMR (400 MHz, $CDCl_3$ and DMSO-d_6). The crude NMR spectrum mainly showed peaks from TMP and tosylate. Most of the signals generated from the salt **11** and amide **6** had disappeared and the desired diaryl ether was not visible (Figure S6).

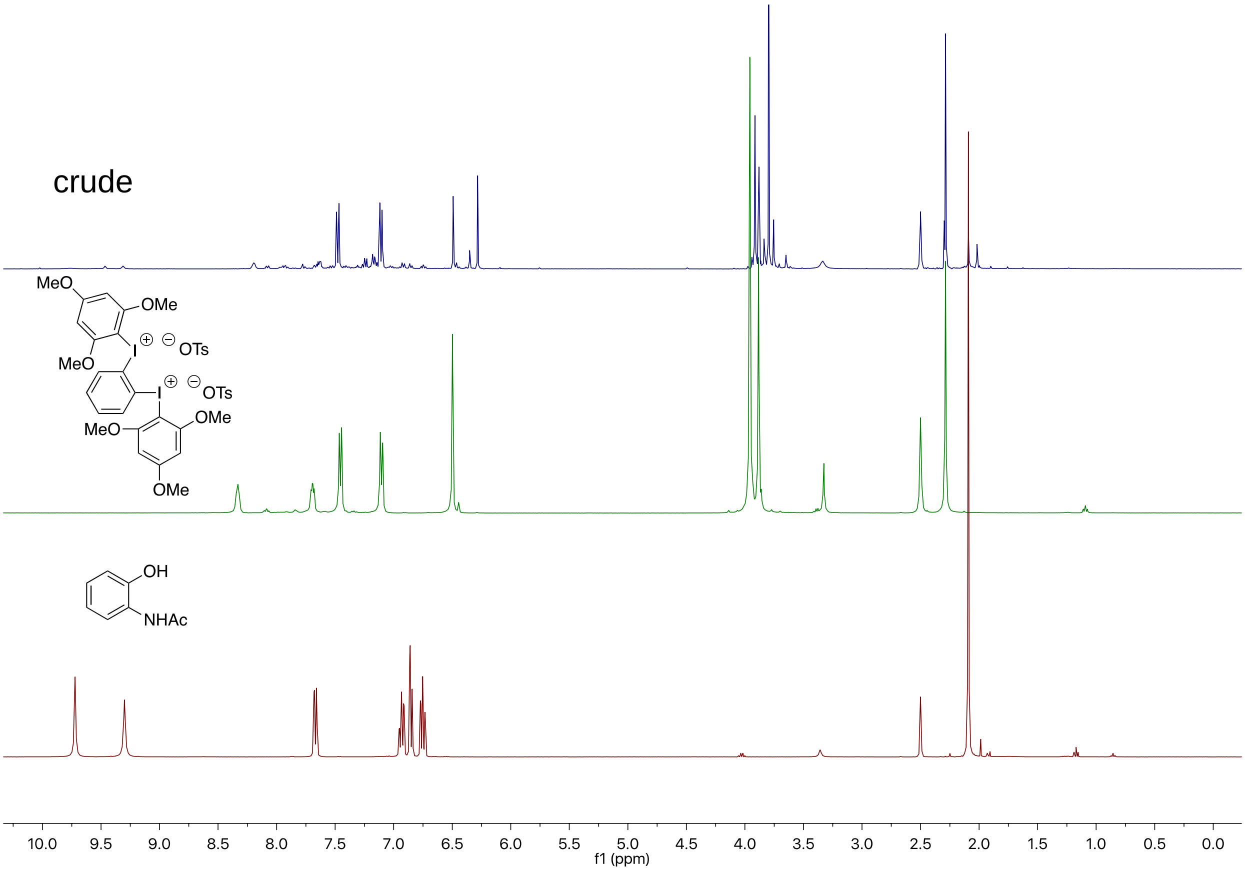

Figure S6. Crude NMR (DMSO-d_6) from the reaction with bisiodonium salt **11**.

6 Cyclization and one-pot reactions

6.1 Cyclization of ether 3

The cyclization was performed according to literature [6], but did not reach completion despite using the same substrate as in literature. The ratio of product **2** to starting material **3** remained roughly 1:1, according to crude ^{1}H NMR analysis, also upon modified conditions (Table S2). Some reactions were directly purified, for which isolated yields are given. The volatility of DMEDA might be the reason for incomplete reactions, but reactions using a larger amount of DMEDA were not superior (entry 3). Microwave vials were examined on small scale, and a screw capped flask was employed in a somewhat larger reaction scale. Still, the reaction might be more successful in larger scale using a smaller screw capped flask.

Table S2. Cyclization reactions.[a]

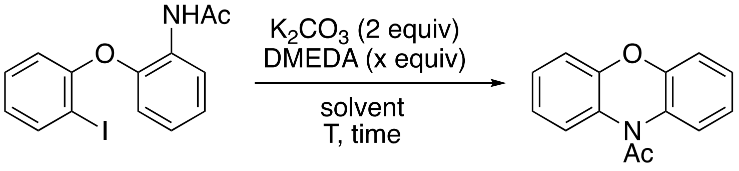

3 **2**

Entry	Solvent	DMEDA (equiv)	Oil bath temp (°C)	Time (h)	Ratio **2**:**3**[b]	Isolated yield **2** (%)	Isolated yield **3** (%)
1[c]	toluene	0.1	135	24	60:40		
2	toluene	0.1	135	72		44	17
3[c]	toluene	1	135	24	40:60		
4[d]	toluene	0.1	135	24		6	93
6	toluene	0.1	135	24		49	49
7	xylene	0.1	145	24	0:100		
8[e]	toluene	0.1	135	24		not detected	not determined

[a] Performed as described below. [b] According to crude ^{1}H NMR. [c] Unpierced septum used in the microwave cap. [d] 1.4 mmol scale performed in screw capped flask (50 mL). [e] *t*-BuOK (2 equiv) was used and base, by-products obtained.

Synthesis of *N*-Acetylphenoxazine (2)

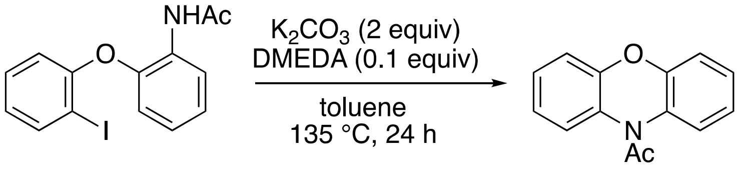

3 **2** 49%
(96% based on recovered **3**)

Diaryl ether **3** (0.10 g, 0.28 mmol, 1 equiv) and K_2CO_3 (0.078 g, 0.57 mmol, 2 equiv) were added to an oven-dried microwave vial (10 mL) that was capped, and argon atmosphere was established. DMEDA (3.05 µL, 0.028 mmol, 0.1 equiv) and toluene (1 mL) were added and the reaction mixture was stirred at 135 °C (oil bath temperature) for 24 h. The reaction was allowed to cool down to room temperature, diluted with CH_2Cl_2 and eluted over a short silica plug. The solution was concentrated under reduced pressure and the crude product was purified via silica gel flash column chromatography (pentane:EtOAc 10:1 → 5:1) to give **2** (0.049 g, 0.139 mmol, 49%) as colorless needle crystals. Unreacted diaryl ether **3** was recovered in 49%, and the yield based on recovered starting material is hence 96% [49/(100-49)]. The analytical data are consistent with previous reports [6].

R_f = 0.44 in pentane:EtOAc 4:1. ^{1}H-NMR (400 MHz, $CDCl_3$): 7.49-7.47 (m, 2 H), 7.22-7.18 (m, 2 H), 7.15-7.10 (m, 4 H), 2.33 (s, 3 H). ^{13}C NMR (101 MHz, $CDCl_3$) δ 169.4, 151.2, 129.7, 127.0, 125.3, 123.5, 117.0, 23.2.

6.2 Attempted one-pot synthesis of *N*-acetylphenoxazine (2)

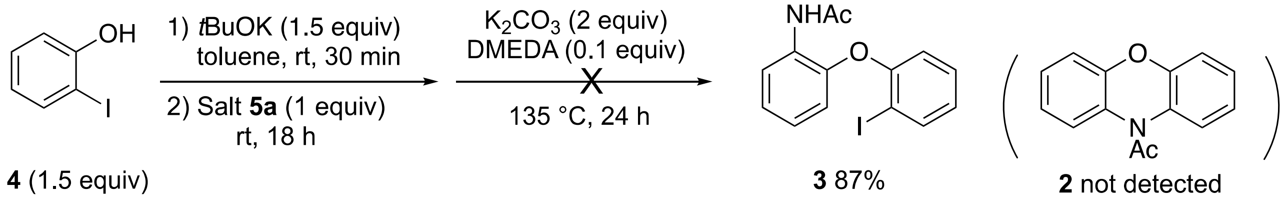

The first step was performed according to the procedure for synthesis of diaryl ether **3** using phenol **4** in 0.2 mmol scale. Then K_2CO_3 (0,055 g, 0,4 mmol, 2 equiv) and DMEDA (2.2 µL, 0.02 mmol, 0.1 equiv) were added under argon flow. The vial was capped again and the reaction mixture was stirred at 135 °C for 24 h. The reaction mixture was cooled down to room temperature and diluted with CH_2Cl_2 and eluted over a short silica plug. The solution was concentrated under reduced pressure to give the crude product. The crude ^{1}H NMR was analyzed using internal standard (see Section 4.2), which showed no formation of the wanted product **2**. Instead, diaryl ether **3** was obtained in 87% yield, indicating that the cyclization step had failed.

7 References

1. Vogel, A. I.; Furniss, B. S.; Hannaford, A. J.; Rogers, V.; Smith, P. W. G.; Tatchell, A. R., *Vogel's Textbook of Practical Organic Chemistry 5th Ed.* Prentice Hall: Harlow, 1996; p 456.
2. Siqueira, F. A.; Taylor, J. G.; Correia, C. R. D. *Tetrahedron Lett.* **2010**, *51*, 2102-2105.
3. Pialat, A.; Liégault, B.; Taillefer, M. *Org. Lett.* **2013**, *15*, 1764-1767.
4. Ozanne-Beaudenon, A.; Quideau, S. *Angew. Chem. Int. Ed.* **2005**, *44*, 7065-7069.
5. Stridfeldt, E.; Lindstedt, E.; Reitti, M.; Blid, J.; Norrby, P.-O.; Olofsson, B. *Chem. Eur. J.* **2017**, 13249-13258.
6. Thomé, I.; Bolm, C. *Org. Lett.* **2012**, *14*, 1892-1895.
