# Supplementary material for: Metal-free formal synthesis of phenoxazine
Source: Beilstein J Org Chem. 2018 Jun 20;14:1491–7. doi: 10.3762/bjoc.14.126 (PMC6036965; doi:10.3762/bjoc.14.126)
Supplement: File 2 — NMR spectra for products 2, 3, 5a, 7a and 11. [file Beilstein_J_Org_Chem-14-1491-s002.pdf]

Supporting Information

for

Metal-free formal synthesis of phenoxazine

Gabriella Kervefors, Antonia Becker, Chandan Dey and Berit Olofsson*

Address: Department of Organic Chemistry, Arrhenius Laboratory, Stockholm University, SE-106-91 Stockholm, Sweden

* Corresponding author

Email: Berit Olofsson - berit.olofsson@su.se

NMR spectra for products 2, 3, 5a, 7a and 11

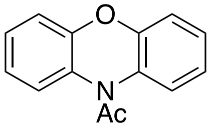

2

^{1}H NMR, $CDCl_3$, 400 MHz

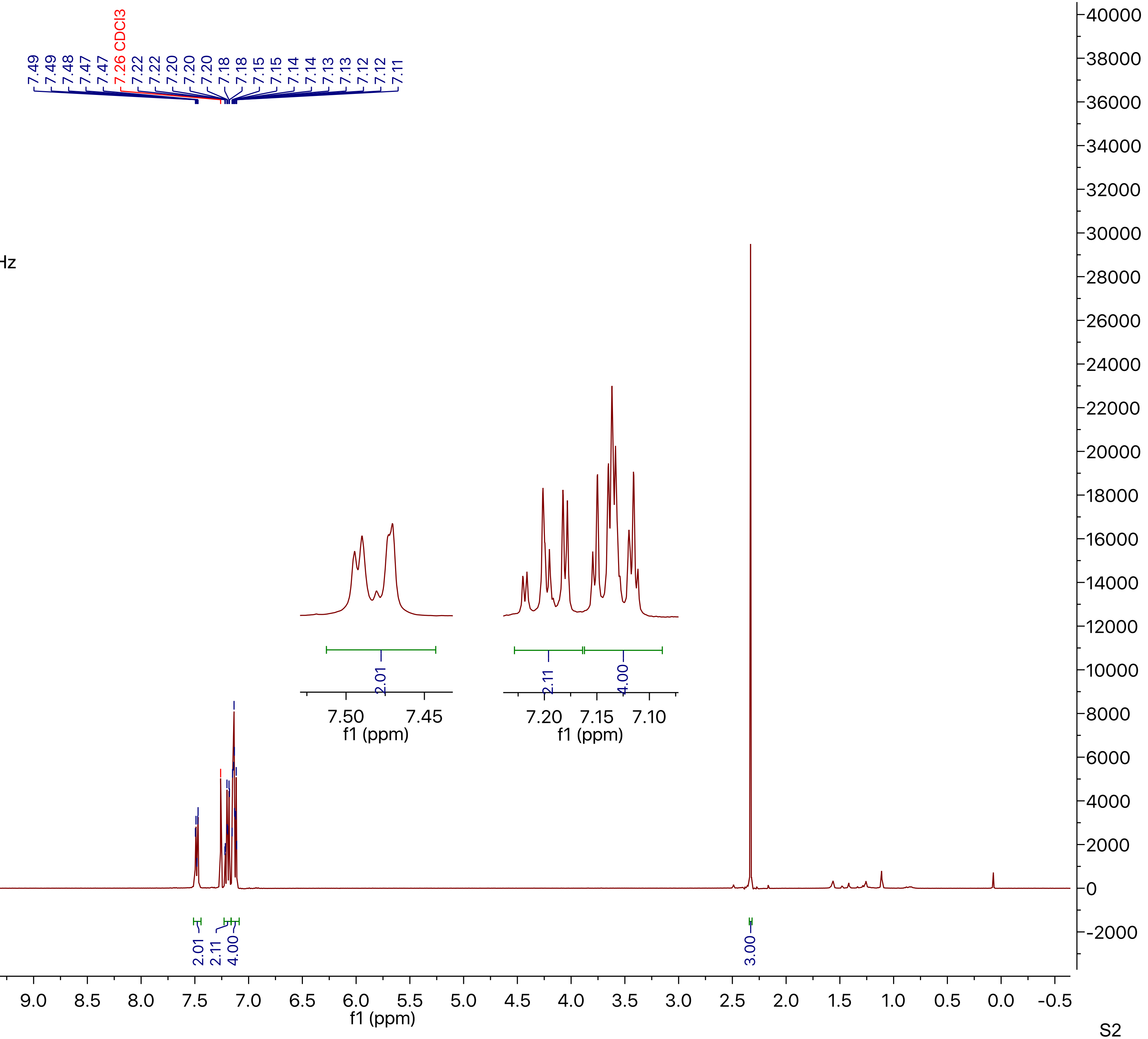

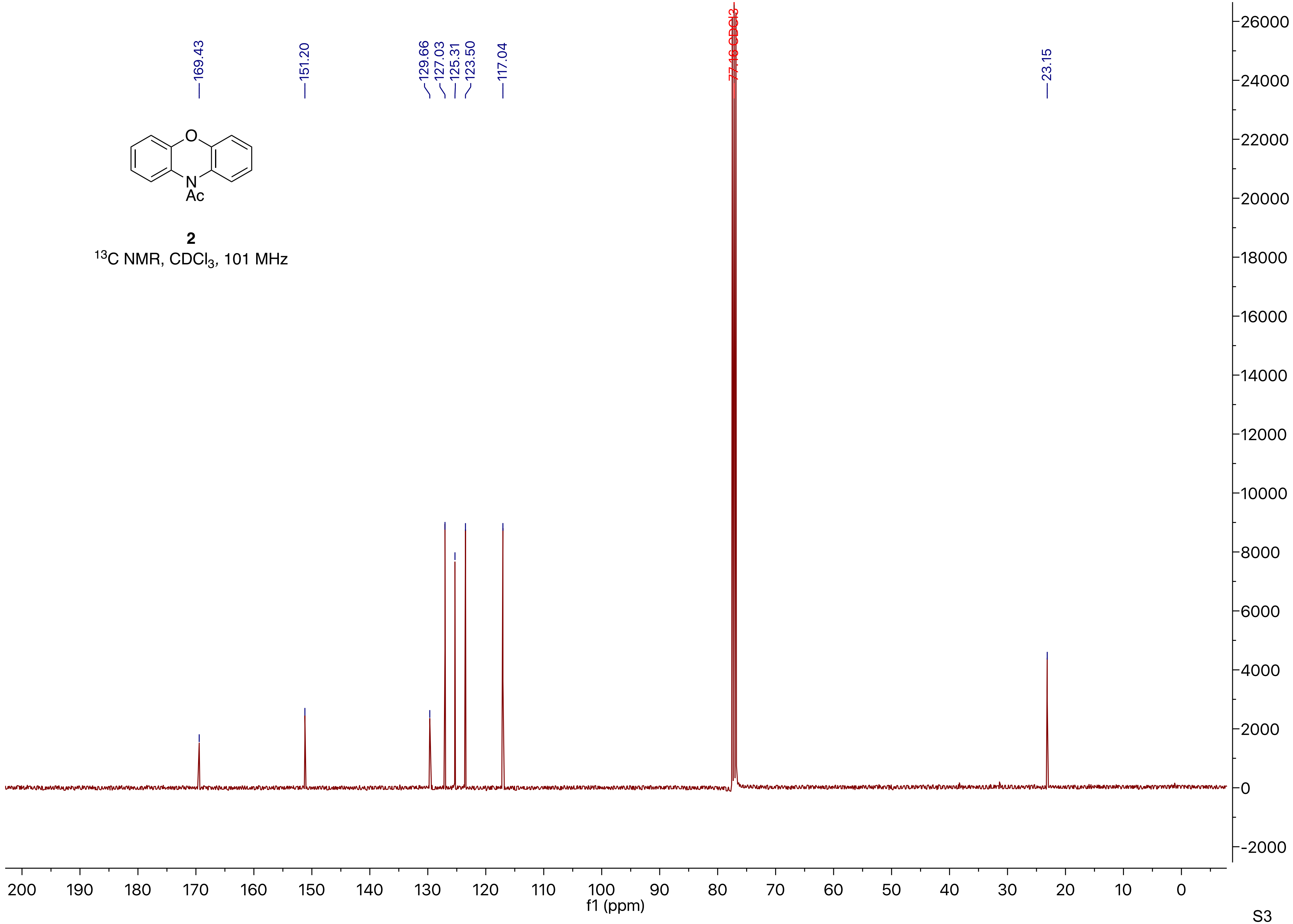
169.43
151.20
129.66
127.03
125.31
123.50
117.04
77.16 CDCl3
23.15
O
N
Ac
2
13C NMR, CDCl3, 101 MHz
26000
24000
22000
20000
18000
16000
14000
12000
10000
8000
6000
4000
2000
0
-2000
200
190
180
170
160
150
140
130
120
110
100
90
80
70
60
50
40
30
20
10
0
f1 (ppm)

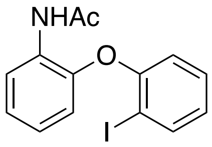

3

^{1}H NMR, $CDCl_3$, 400 MHz

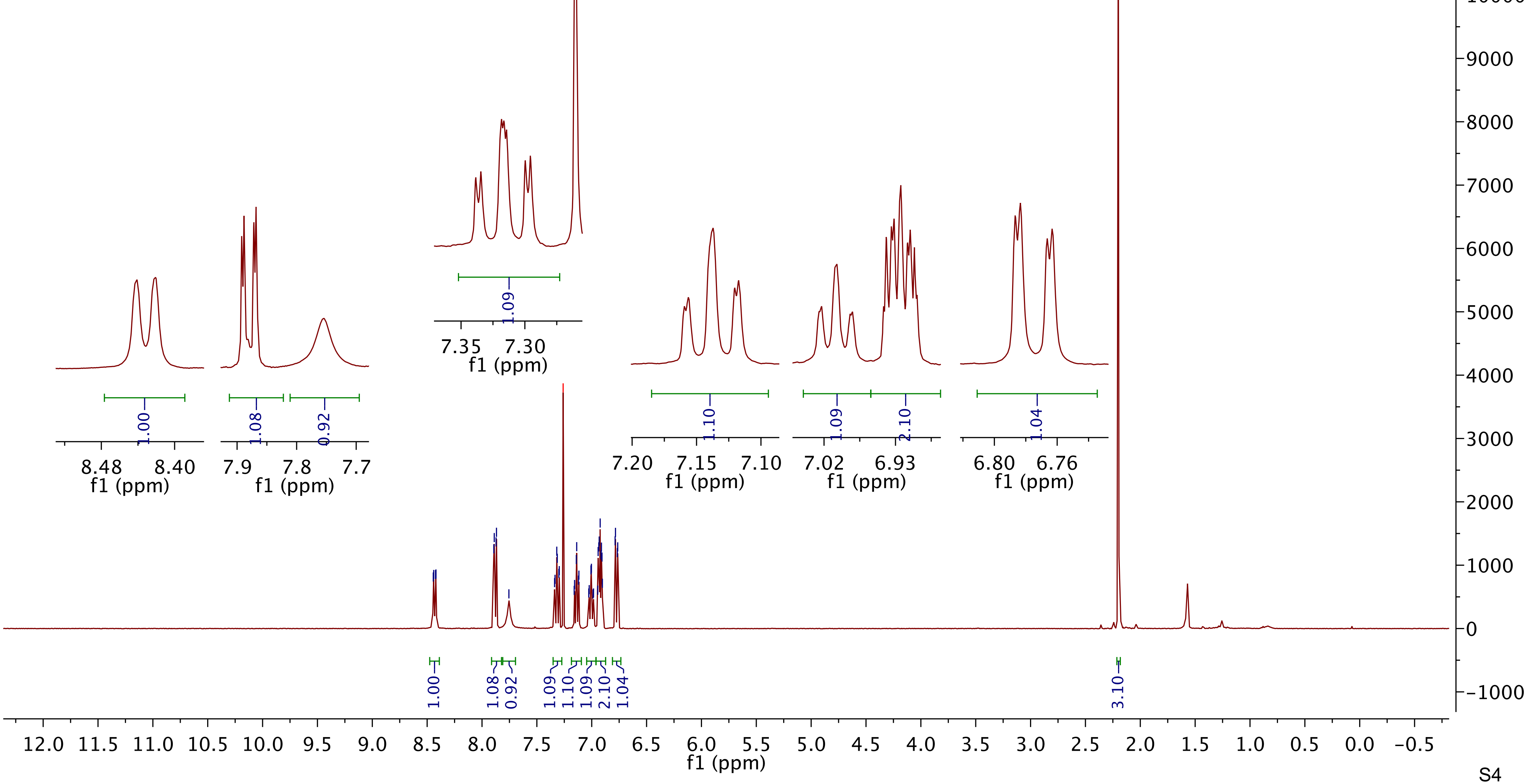

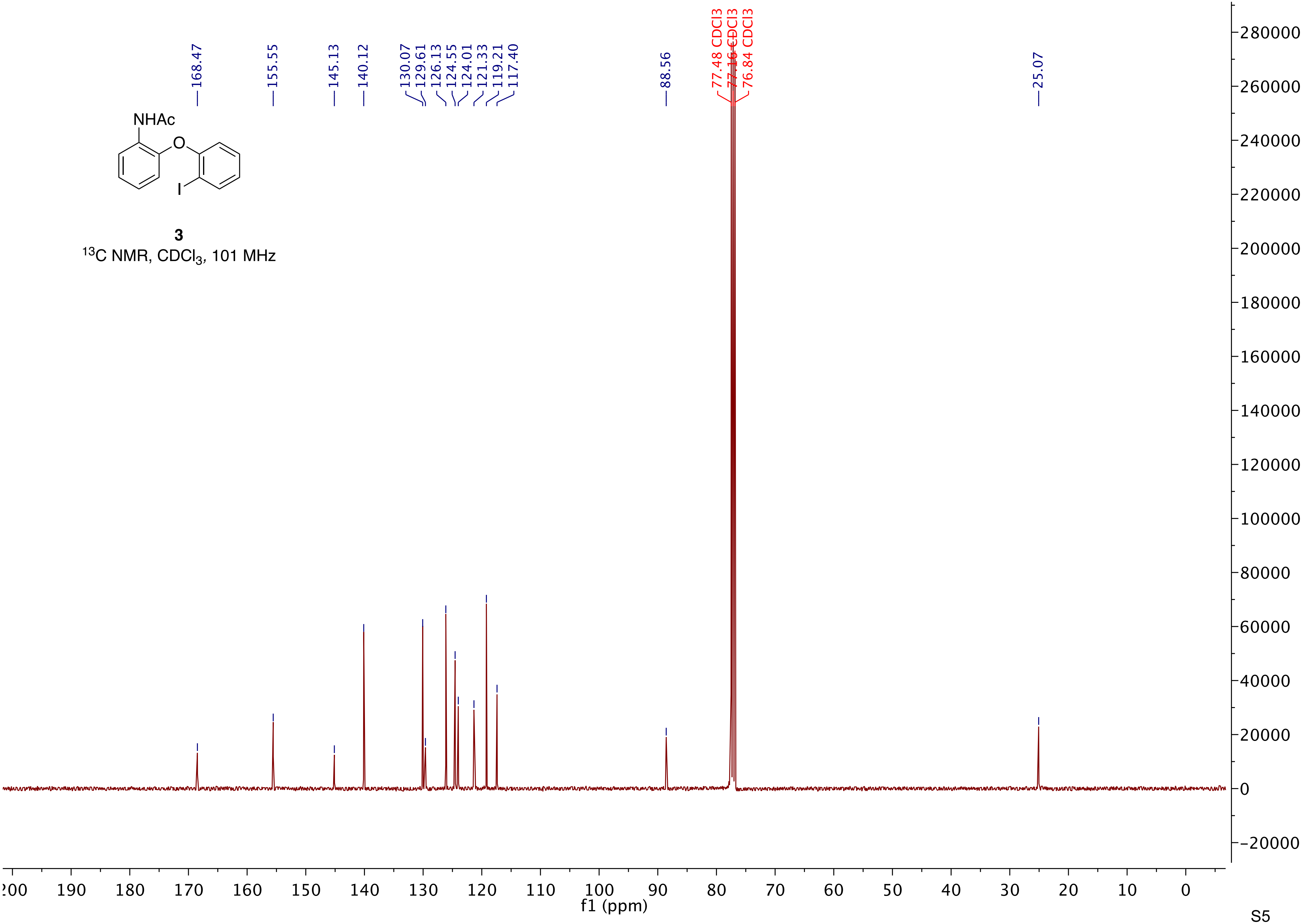

3

^{13}C NMR, $CDCl_3$, 101 MHz

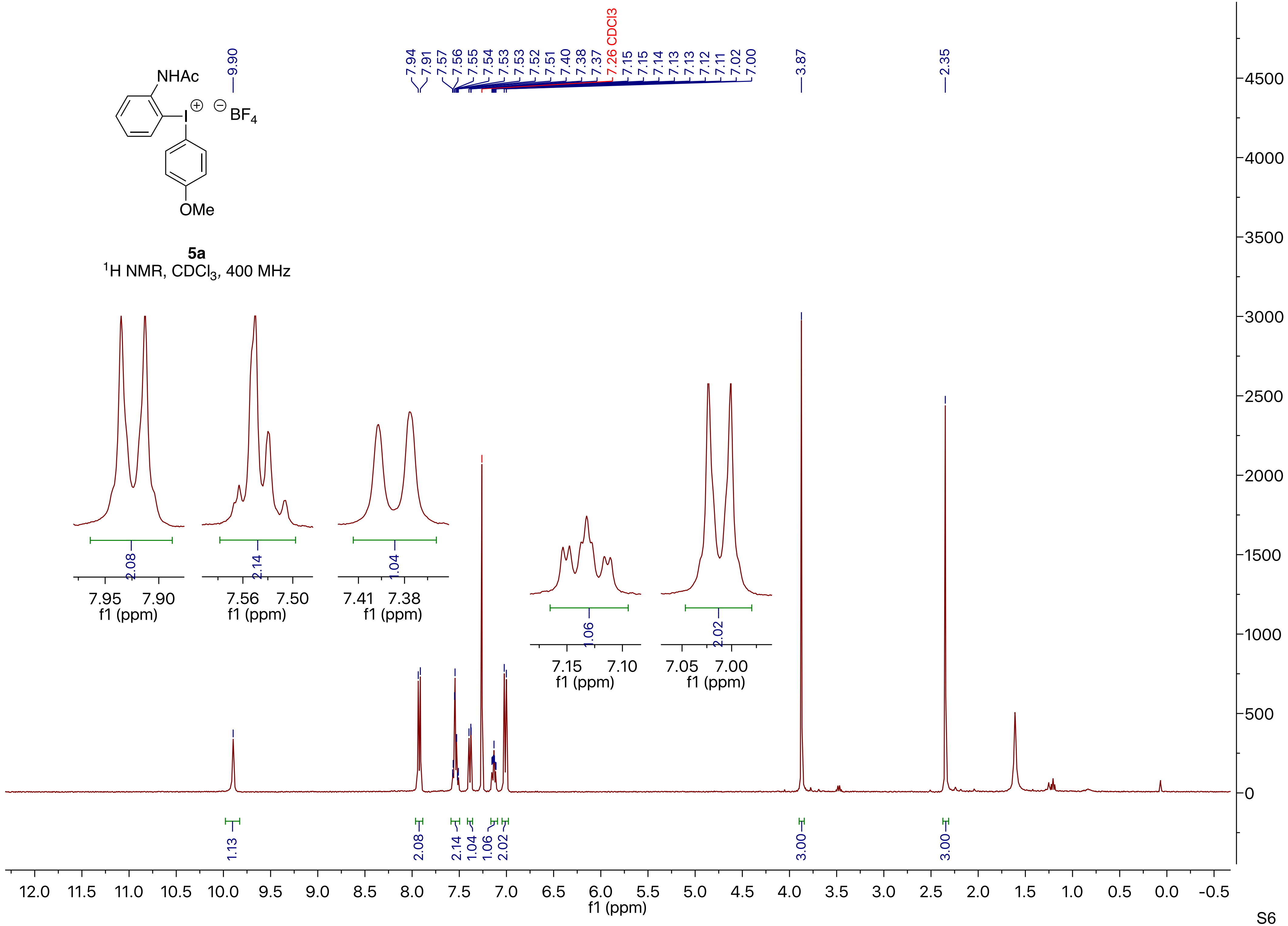

NHAc
BF4
OMe
5a
1H NMR, CDCl3, 400 MHz
9.90
7.94
7.91
7.57
7.56
7.55
7.54
7.53
7.53
7.52
7.51
7.40
7.38
7.37
7.26 CDCl3
7.15
7.15
7.14
7.13
7.13
7.12
7.11
7.02
7.00
3.87
2.35
1.13
2.08
2.14
1.04
1.06
2.02
3.00
3.00
f1 (ppm)

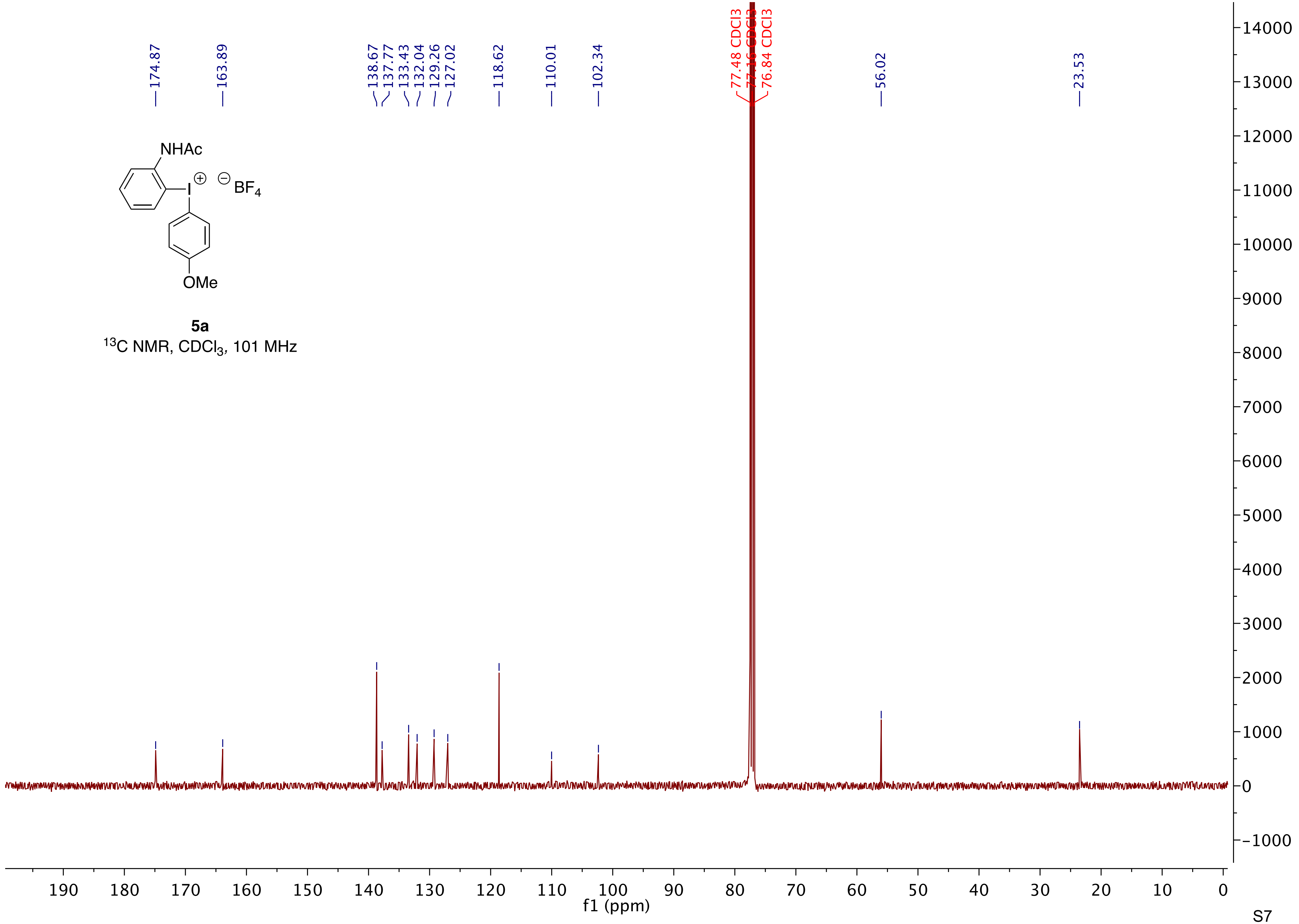
174.87
163.89
138.67
137.77
133.43
132.04
129.26
127.02
118.62
110.01
102.34
77.48 CDCl3
77.16 CDCl3
76.84 CDCl3
56.02
23.53
NHAc
I
BF4
OMe
5a
13C NMR, CDCl3, 101 MHz
f1 (ppm)

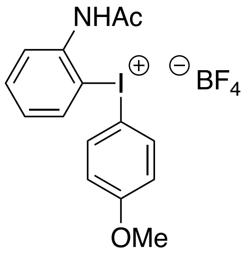

5a

^{19}F NMR, $CDCl_3$, 377 MHz

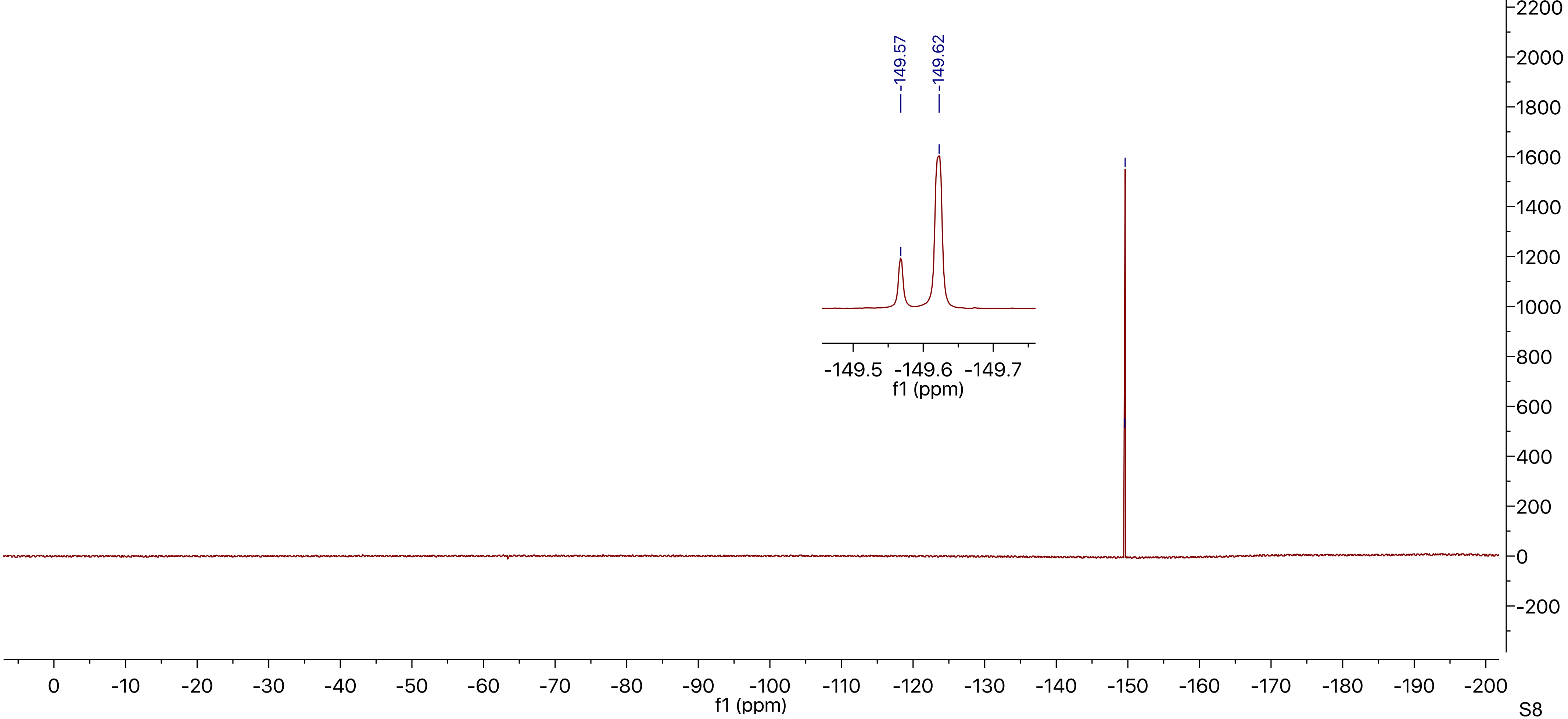

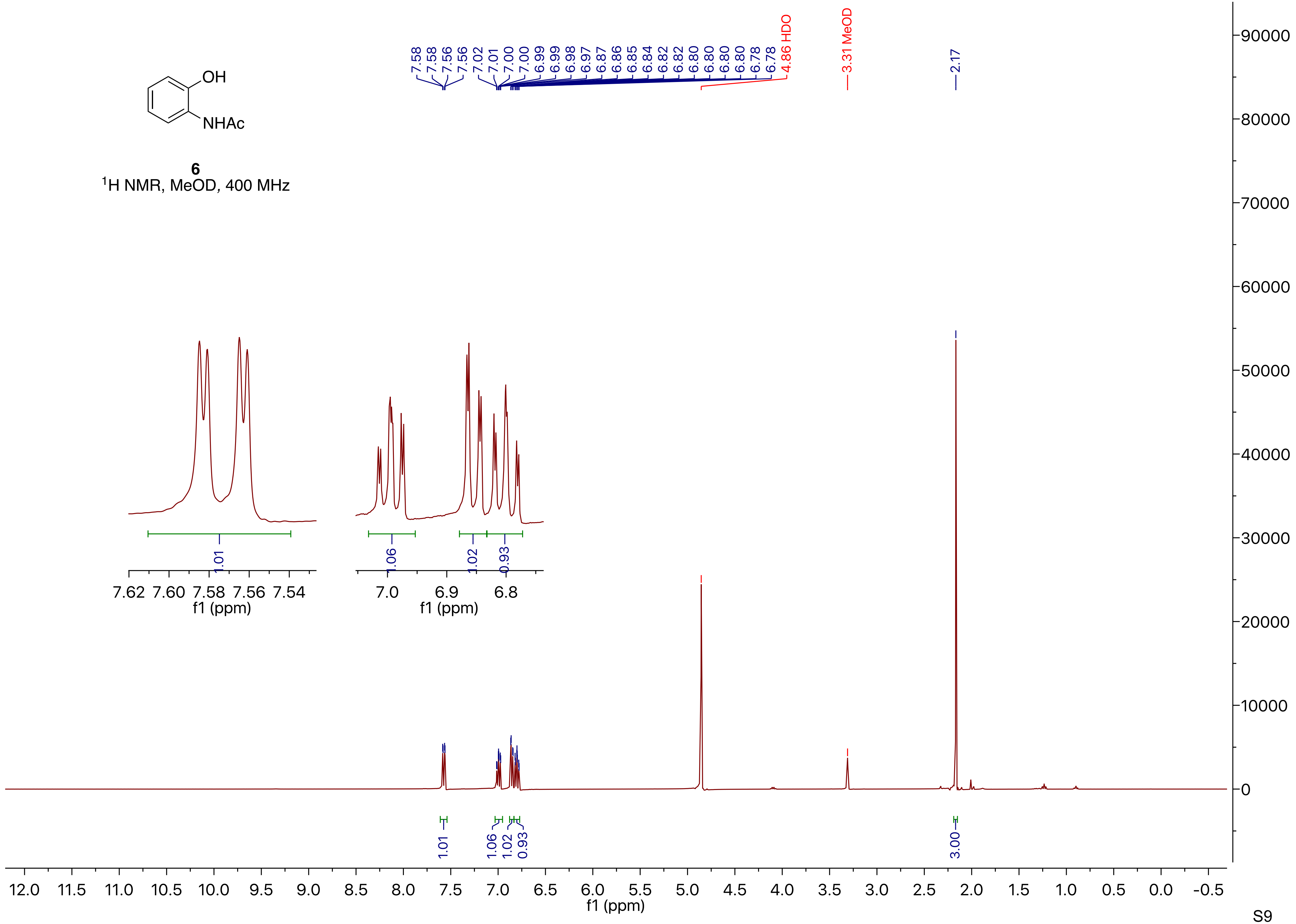
OH
NHAc
6
1H NMR, MeOD, 400 MHz
7.58
7.58
7.56
7.56
7.02
7.01
7.00
7.00
6.99
6.99
6.98
6.97
6.87
6.86
6.85
6.84
6.82
6.82
6.80
6.80
6.80
6.80
6.78
6.78
4.86 HDO
3.31 MeOD
2.17
1.01
1.06
1.02
0.93
3.00
f1 (ppm)

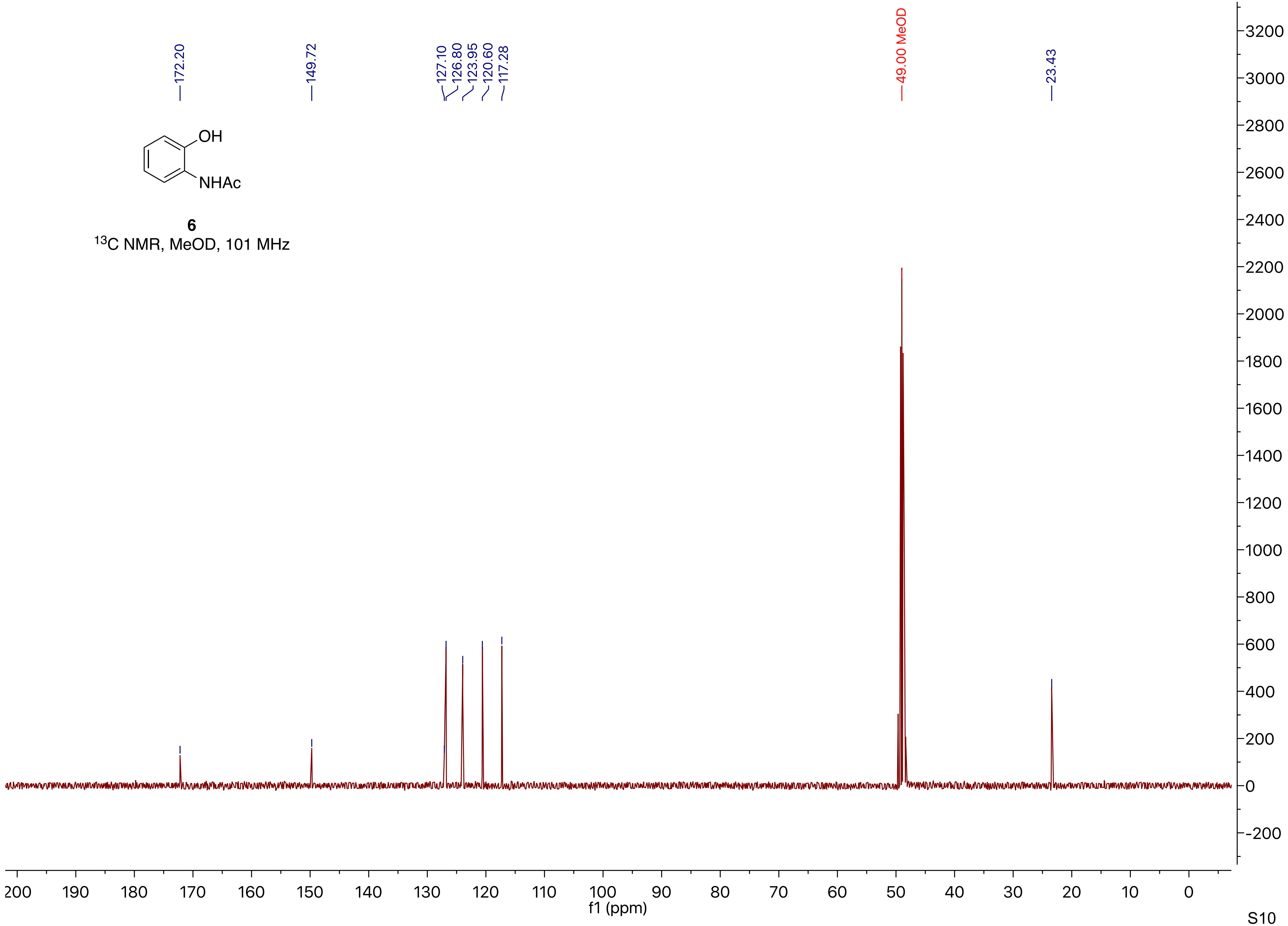
172.20
149.72
127.10
126.80
123.95
120.60
117.28
49.00 MeOD
23.43
OH
NHAc
6
^{13}C NMR, MeOD, 101 MHz
f1 (ppm)

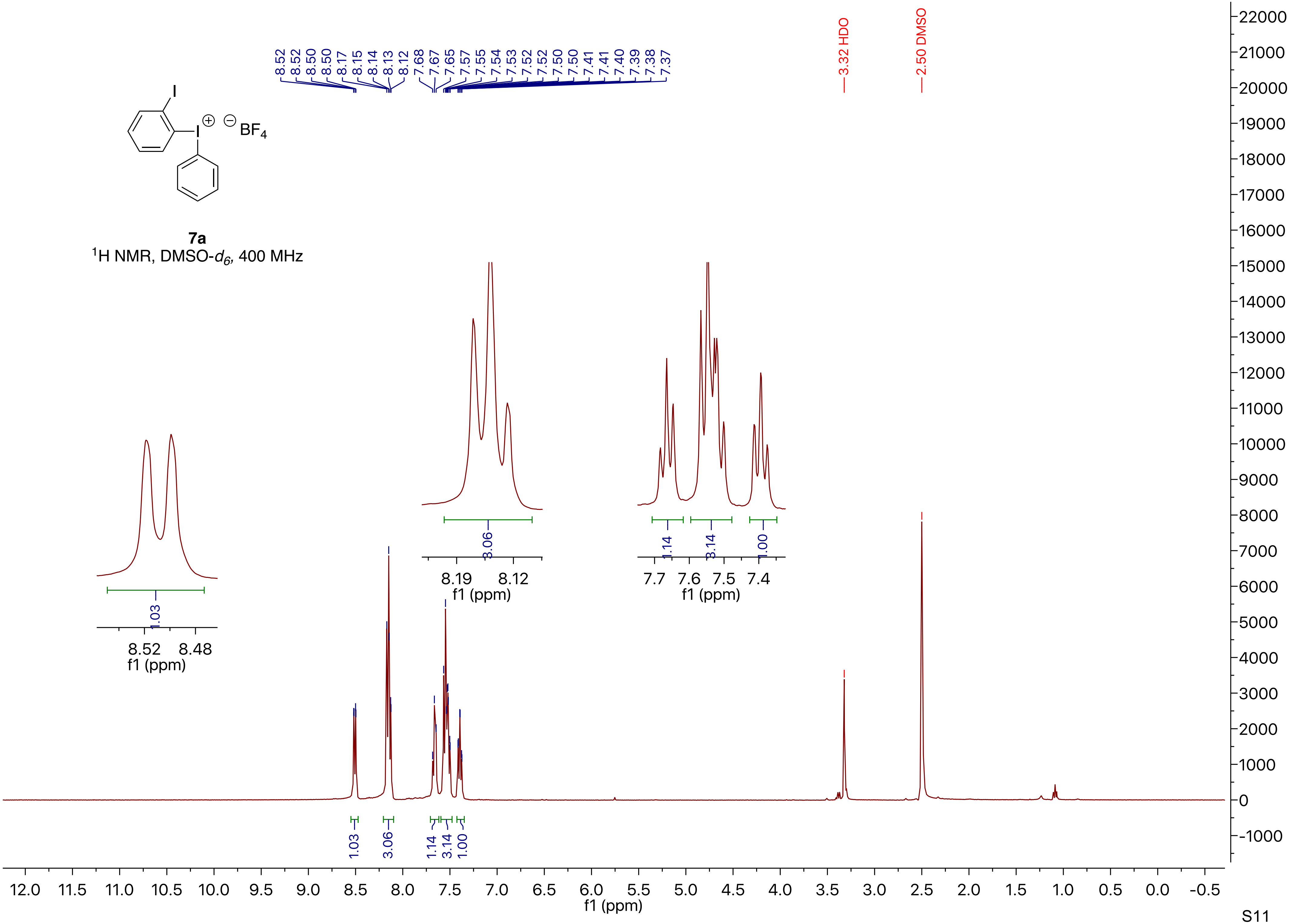

I
BF4
7a
1H NMR, DMSO-d6, 400 MHz
8.52 8.52 8.50 8.50 8.17 8.15 8.14 8.13 8.12 7.68 7.67 7.65 7.57 7.55 7.54 7.53 7.52 7.52 7.50 7.50 7.41 7.41 7.40 7.39 7.38 7.37
3.32 HDO
2.50 DMSO
1.03
3.06
1.14
3.14
1.00
8.52 8.48
8.19 8.12
7.7 7.6 7.5 7.4
f1 (ppm)
12.0 11.5 11.0 10.5 10.0 9.5 9.0 8.5 8.0 7.5 7.0 6.5 6.0 5.5 5.0 4.5 4.0 3.5 3.0 2.5 2.0 1.5 1.0 0.5 0.0 -0.5
22000 21000 20000 19000 18000 17000 16000 15000 14000 13000 12000 11000 10000 9000 8000 7000 6000 5000 4000 3000 2000 1000 0 -1000

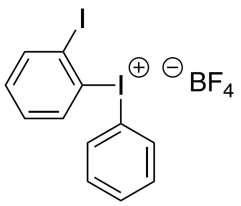

7a

^{13}C NMR, DMSO-d_6, 101 MHz

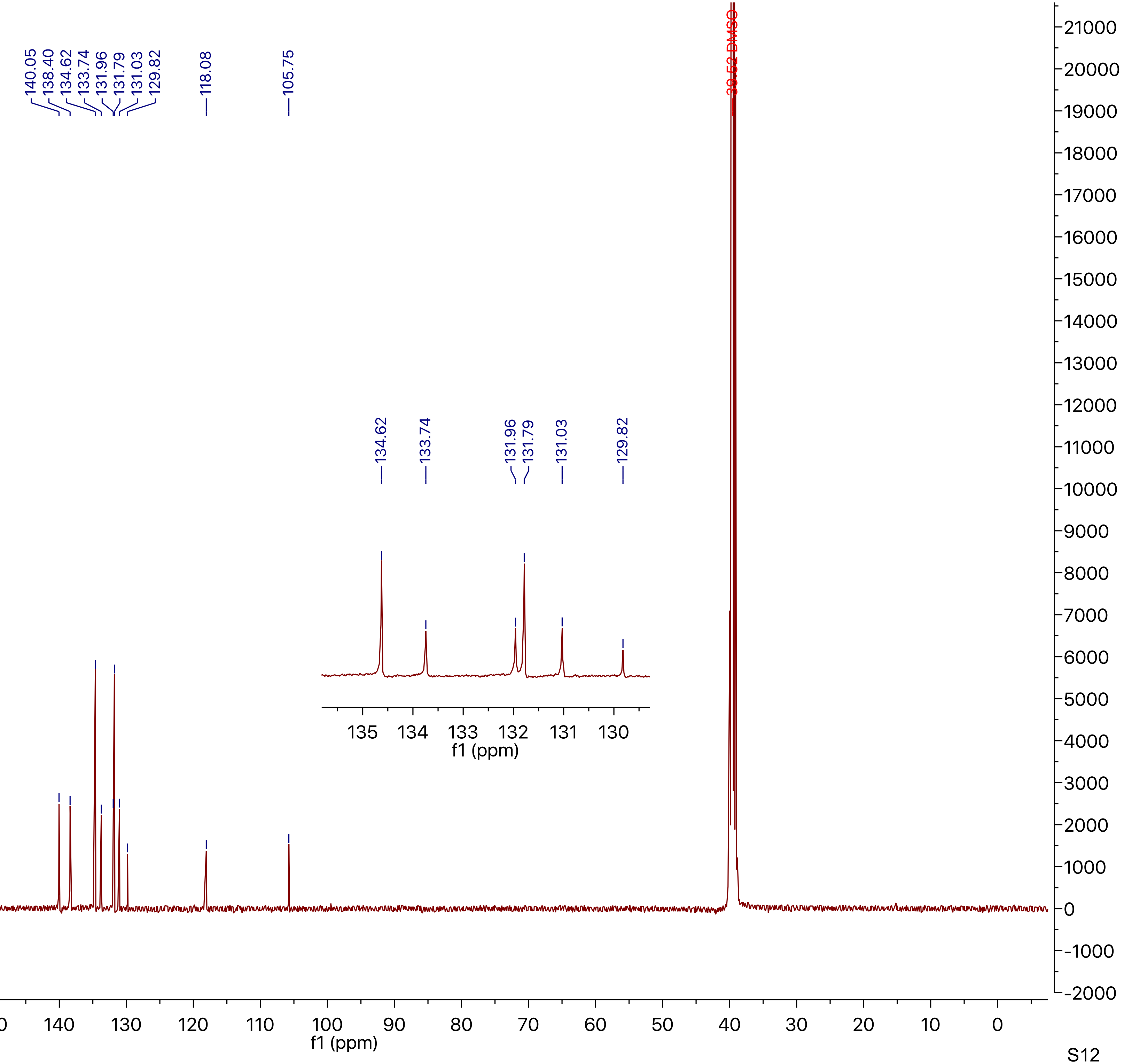

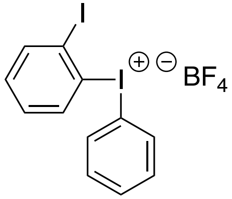

7a
^{19}F NMR, DMSO-d_6, 377 MHz

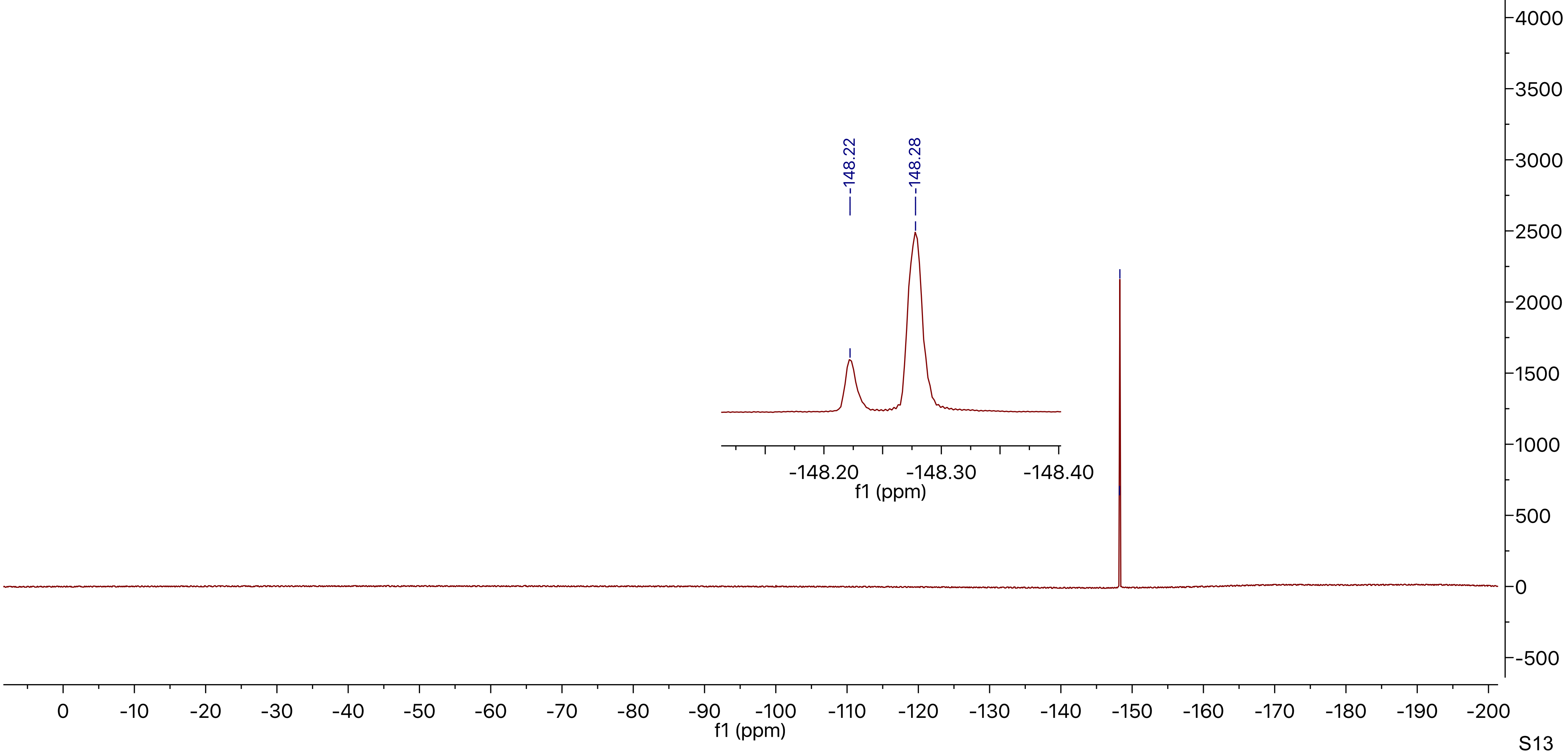

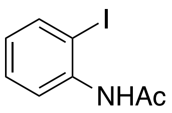

8

^1H NMR, $CDCl_3$, 400 MHz

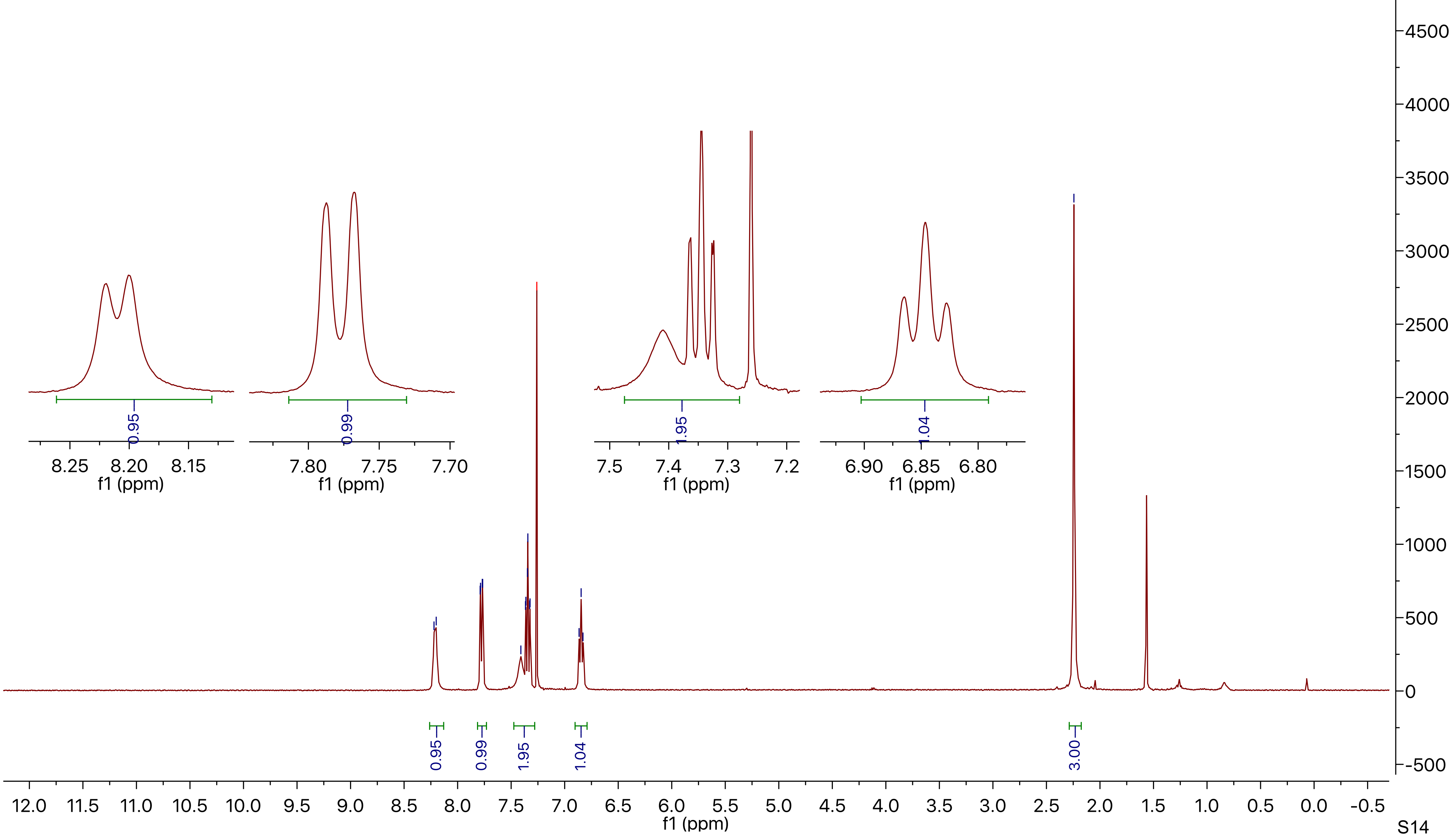

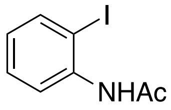

8

^{13}C NMR, $CDCl_3$, 101 MHz

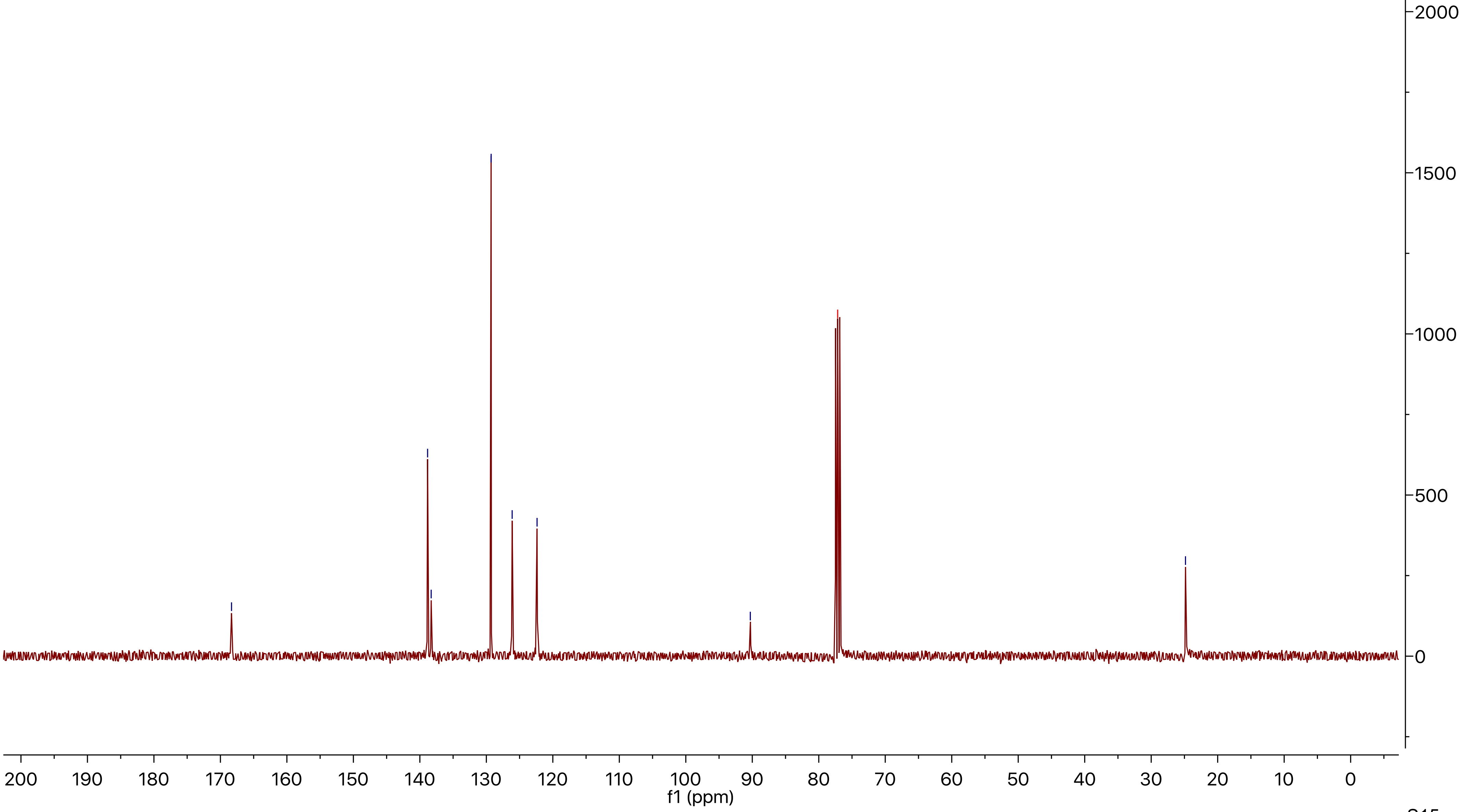

COSY of crude reaction with 3:9 ratio of 1:0.7

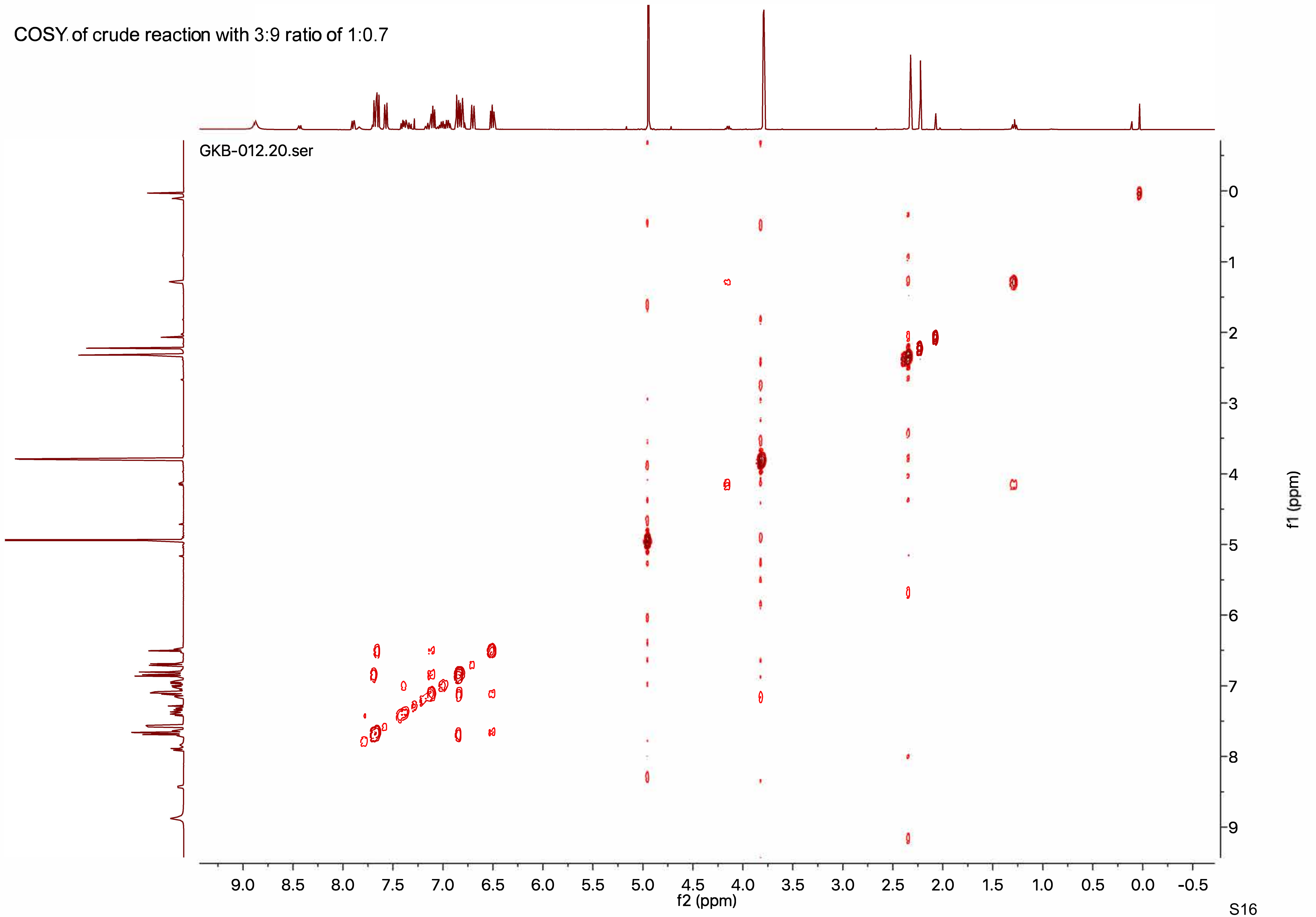

COSY of crude reaction with **3:9** ratio of 1:0.7

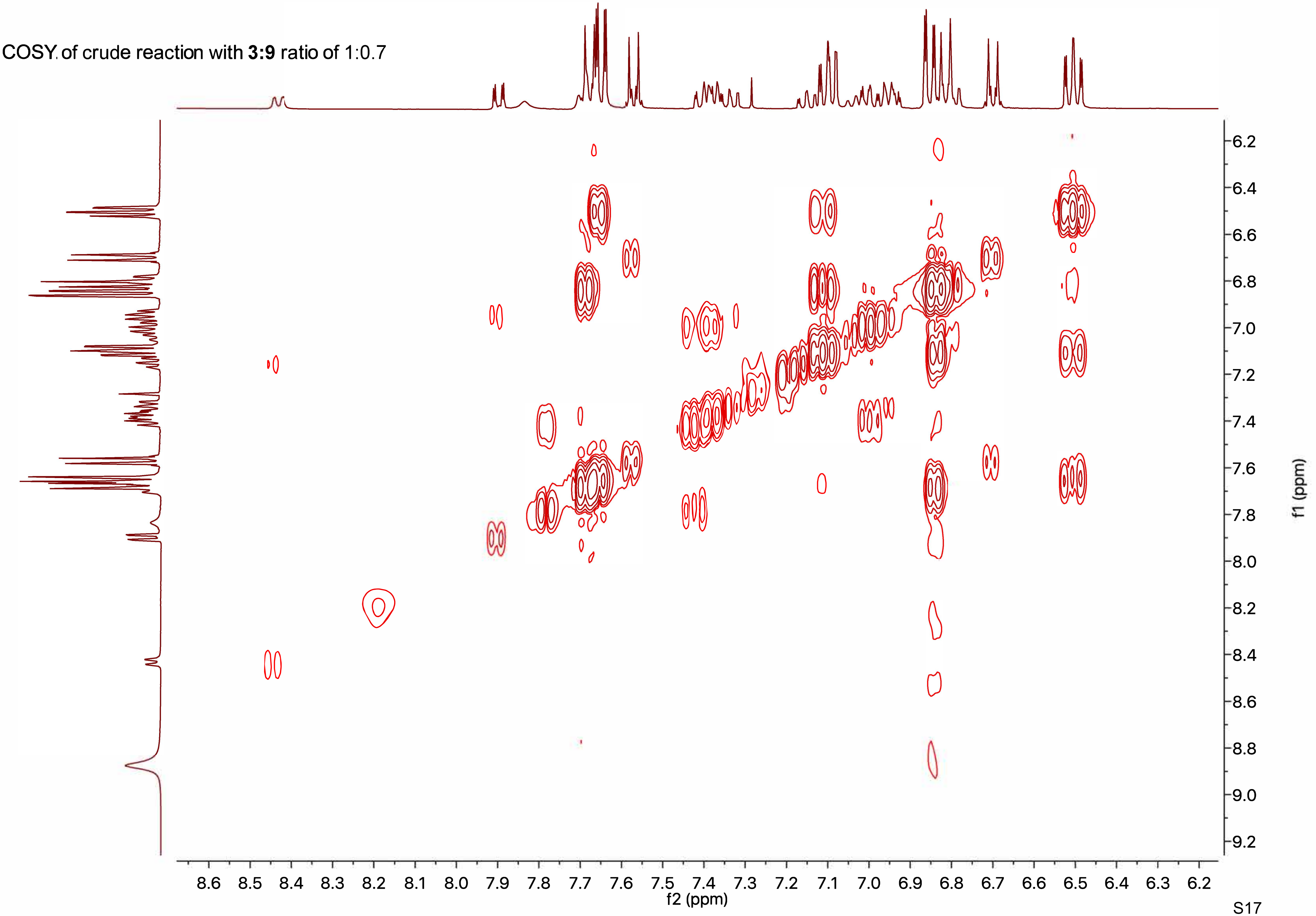

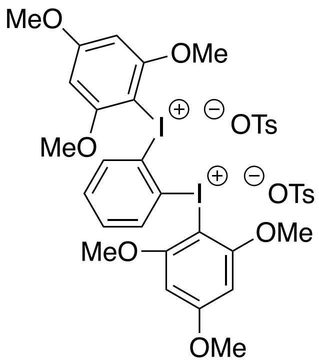

11
^{1}H NMR, DMSO-d_6, 400 MHz

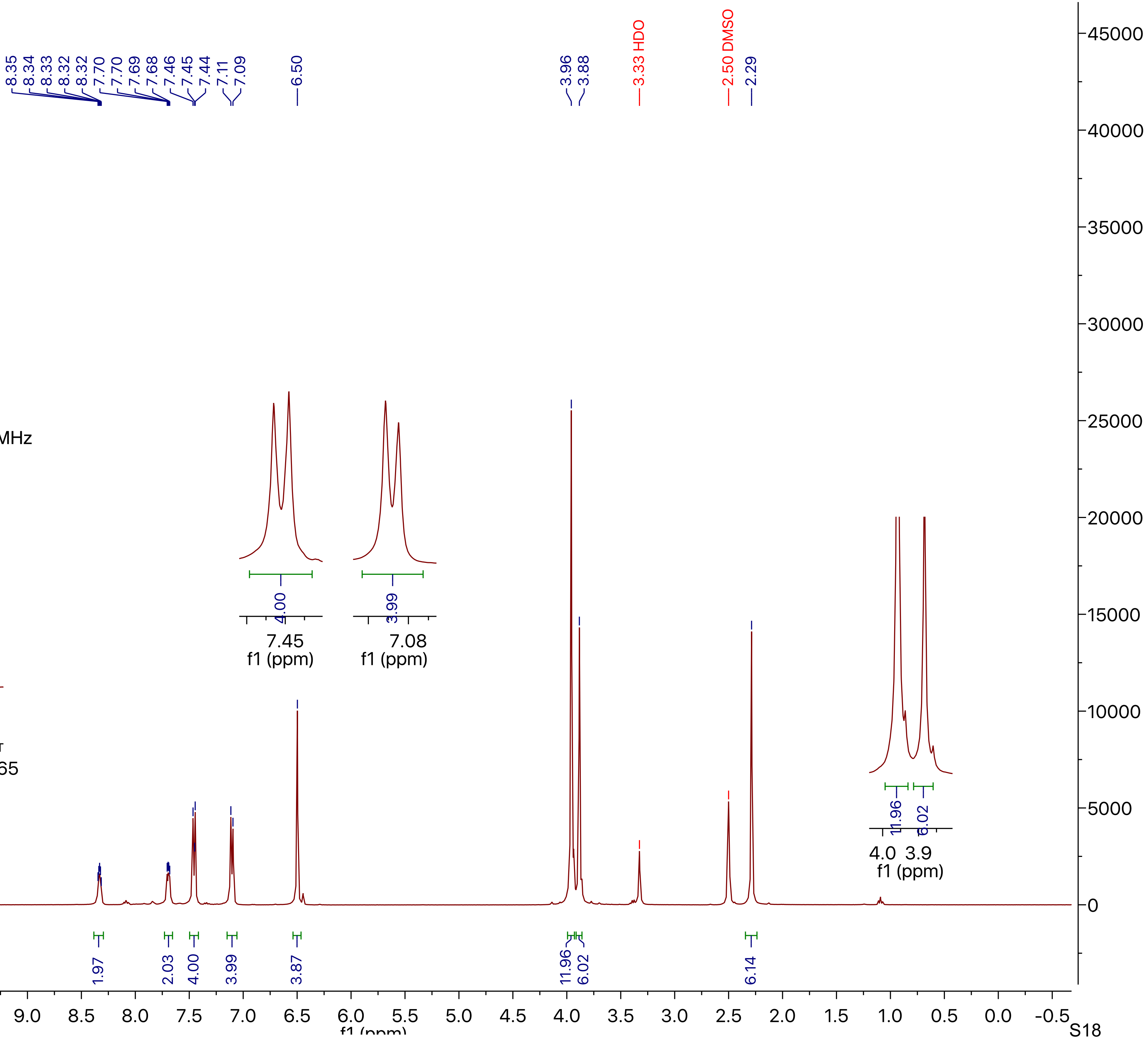

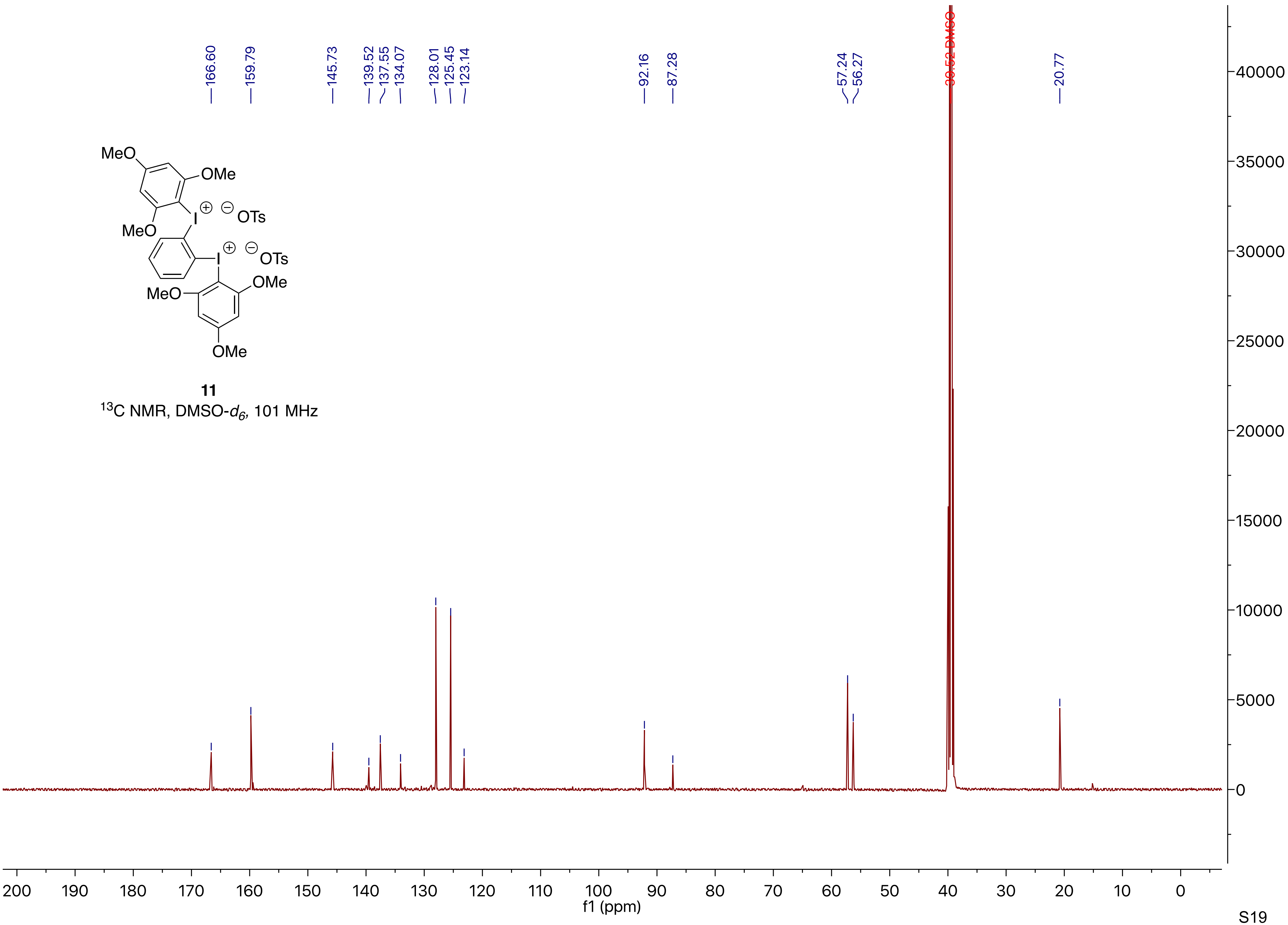
166.60
159.79
145.73
139.52
137.55
134.07
128.01
125.45
123.14
92.16
87.28
57.24
56.27
39.52 DMSO
20.77
MeO
OMe
OTs
MeO
OTs
OMe
MeO
OMe
11
^{13}C NMR, DMSO-d_6, 101 MHz
f1 (ppm)

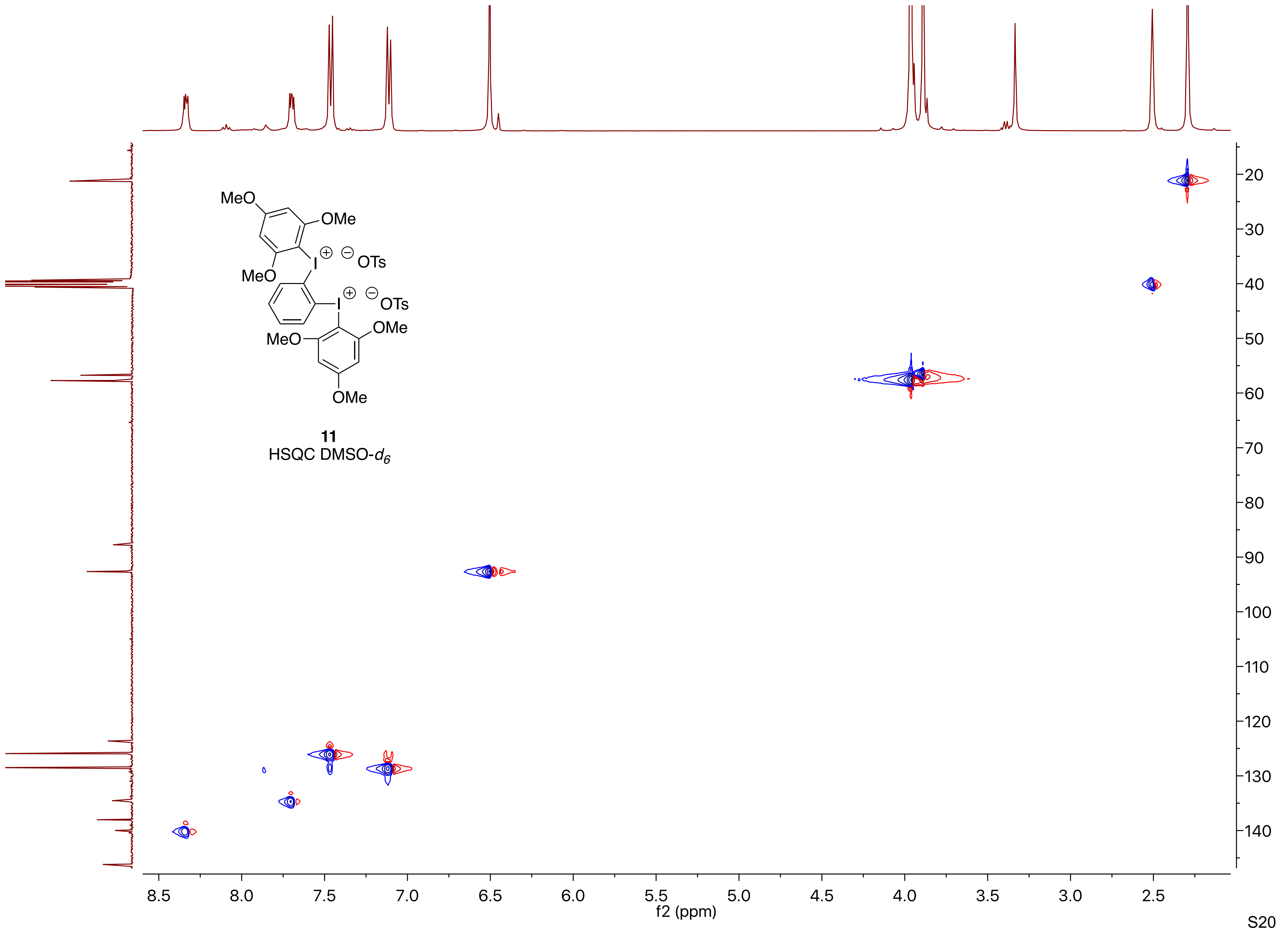
MeO
OMe
OTs
MeO
OTs
MeO
OMe
OMe
11
HSQC DMSO-d_6
f2 (ppm)
f1 (ppm)
8.5 8.0 7.5 7.0 6.5 6.0 5.5 5.0 4.5 4.0 3.5 3.0 2.5
20 30 40 50 60 70 80 90 100 110 120 130 140
